# Regulatory feedback response mechanisms to phosphate starvation in rice

**DOI:** 10.1038/s41540-017-0041-0

**Published:** 2018-01-08

**Authors:** Ishan Ajmera, Jing Shi, Jitender Giri, Ping Wu, Dov J. Stekel, Chungui Lu, T. Charlie Hodgman

**Affiliations:** 1 School of Biosciences, The University of Nottingham, Sutton Bonington Campus, Sutton Bonington, Loughborough, LE12 5RD UK; 2 Centre for Plant Integrative Biology, University of Nottingham, Sutton Bonington, Loughborough, LE12 5RD UK; 3 State Key Laboratory of Plant Physiology and Biochemistry, College of Life Science, Zhejiang University, Hangzhou, China; 4 Department of Biology, Texas A&M University, College Station, TX USA; 5 National Institute of Plant Genome Research, Aruna Asaf Ali Marg, New Delhi, India; 6 School of Animal, Rural and Environmental Sciences, Nottingham Trent University, Nottingham, NG1 4FQ UK

## Abstract

Phosphorus is a growth-limiting nutrient for plants. The growing scarcity of phosphate stocks threatens global food security. Phosphate-uptake regulation is so complex and incompletely known that attempts to improve phosphorus use efficiency have had extremely limited success. This study improves our understanding of the molecular mechanisms underlying phosphate uptake by investigating the transcriptional dynamics of two regulators: the Ubiquitin ligase PHO2 and the long non-coding RNA IPS1. Temporal measurements of RNA levels have been integrated into mechanistic mathematical models using advanced statistical techniques. Models based solely on current knowledge could not adequately explain the temporal expression profiles. Further modeling and bioinformatics analysis have led to the prediction of three regulatory features: the PHO2 protein mediates the degradation of its own transcriptional activator to maintain constant PHO2 mRNA levels; the binding affinity of the transcriptional activator of *PHO2* is impaired by a phosphate-sensitive transcriptional repressor/inhibitor; and the extremely high levels of IPS1 and its rapid disappearance upon Pi re-supply are best explained by Pi-sensitive RNA protection. This work offers both new opportunities for plant phosphate research that will be essential for informing the development of phosphate efficient crop varieties, and a foundation for the development of models integrating phosphate with other stress responses.

## Systems biology for sustainability: models for phosphate uptake regulation in plants

Food security is a global priority. One aspect of this is the ability to grow crops in poorer soils with less fertilizer input, of which phosphate is both essential and resource limited. This study provides a quantitative understanding of the genetic regulation of phosphate uptake in rice upon its deficiency. The mathematical models developed in this article lead to three hypotheses for the gaps identified in current knowledge. One of these hypotheses has previously only been reported in animals while the other prompted laboratory experiments, revealing an extra level of regulation at short timescales. These models provide the basis for crop systems biologists to study other aspects of phosphate regulation, including its internal utilisation, external availability and foraging, and, more crucially, in response to other stresses.

## Introduction

Ensuring a secure and sustainable food supply for the growing human population is a global priority. Current agricultural production is unsustainable because of the increasing scarcity of fresh water, limited availability of land, global climate change, degradation of soil and the depletion of fertilizer stocks. Phosphate availability is often the limiting factor in crop growth, and its interactions with other nutrients are complex.^1–3^ The use of phosphate fertilizer is both economically and ecologically unsustainable, because phosphate stocks are a non-renewable mineral resource,^4^ and the run-off of surplus fertilizer damages the environment.^5^ An alternative to the high use of phosphate fertilizer is to develop crops with increased phosphorus use efficiency,^6,7^ though success on this front has been hampered by the complexity of phosphate chemistry and plant responses to the low availability of soil phosphate.^8^ It is essential to better understand the mechanisms involved in regulating phosphate uptake and homeostasis in plants.

Inorganic phosphate (Pi) starvation triggers a broad range of adaptive responses at the biochemical, genetic, physiological, morphological, anatomical and rhizospheric scales. These include derepression of high-affinity phosphate transporters on the outer cell membranes, a metabolic shift to use of sulfate, reduced photosynthesis, reduced shoot growth, altered root architecture (including lateral root formation and long root hairs), formation of aerenchyma, and secretion of exudates into the surrounding soil to solubilize phosphate, stimulate soil bacteria and attract filamentous fungi to receive Pi from long distances. Pi-starvation responses (PSRs) are themselves complex, but regulated by mechanisms largely conserved between Arabidopsis and rice.^9^

The key transcriptional activator (TA) in Pi starvation signaling is phosphate starvation response 1 in Arabidopsis, and its ortholog OsPHR2 in rice (*Oryza sativa*), see Supplementary Fig. 1. The gene for the latter is constitutively expressed, but the protein does not become active until it is both free to migrate to the nucleus (which occurs with the proteolysis of SPX4 under low cytosolic Pi conditions),^10^ and be sumoylated (which is a general feature of plant abiotic stress).^11^ The active form results in the expression of numerous Pi-starvation-induced genes, including some to make better use of the current cellular phosphate and a microRNA, miR399, which is the systemic integrator defining Pi demand across the whole plant. Mostly miR399 is expressed in shoots and translocated to the root via the phloem, causing degradation of PHO2 mRNA through target mimicry. The *PHO2* gene encodes a ubiquitin-conjugating E2 enzyme (UBC24) that indirectly inhibits high-affinity Pi transporters (PHTs) and phosphate 1 (PHO1), the transporter which puts Pi into the xylem for systemic distribution.^12–16^ The PHO2 mRNA-miRNA399 interaction is also modulated by a long non-coding RNA, At4 in Arabidopsis and induced by phosphate starvation 1 (IPS1) in rice.^17,18^ *OsIPS1* is induced by Pi starvation and has a region partially complementary to miR399,^19^ which enables it to sequester miR399, reducing the availability of free miR399 for degradation and inhibiting complete silencing of PHO2 mRNA.

This work integrates mathematical modeling, informatics and laboratory techniques to analyze the molecular regulation of phosphate acquisition during deficiency, with particular emphasis on PHO2 and IPS1 transcript dynamics. Three new regulatory features are predicted: first, that factors other than miR399 are responsible for the early decline in PHO2 transcript levels; second, that *PHO2 *appears to negatively regulate its own expression; and third, that the high levels of IPS1 and its rapid drop upon Pi re-supply are best explained by Pi-sensitive RNA protection (RP). The resulting mathematical model also provides a base for studying other aspects of the phosphate-starvation response, including its combined effect with other stresses.

## Results

### Delayed expression of miR399 compared with PHO2 mRNA loss

**Fig. 1:**
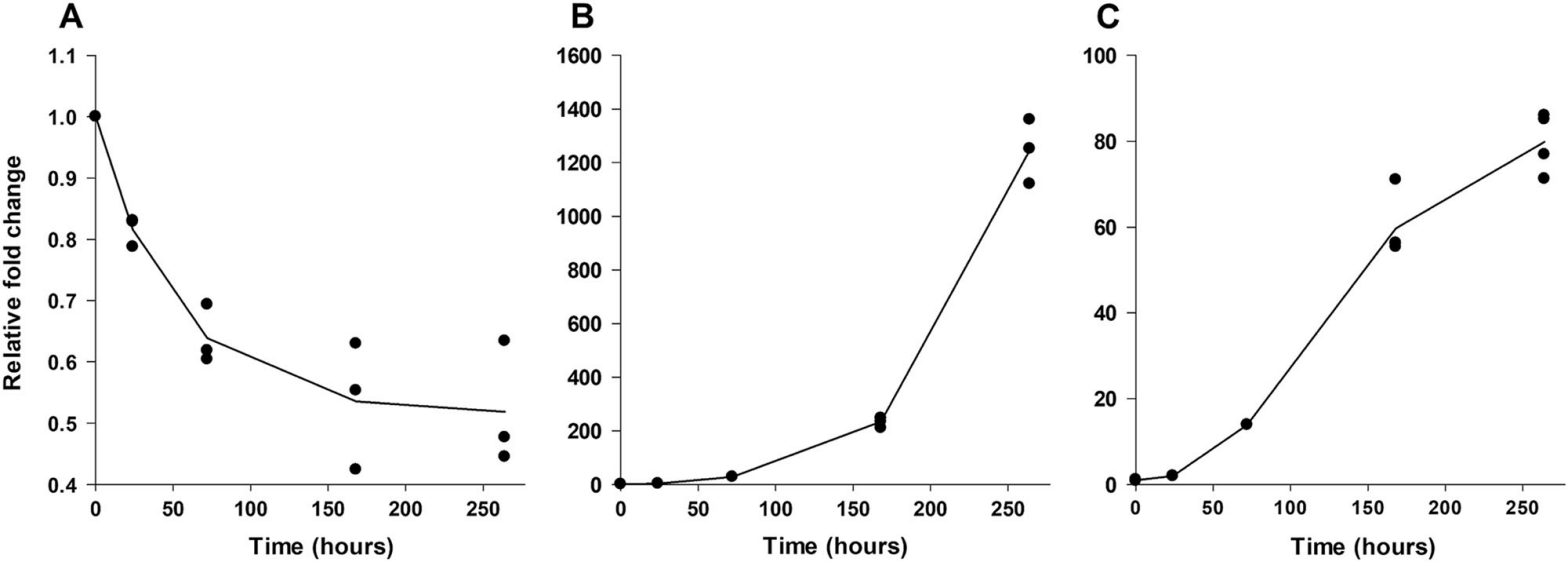
Expression profiles of **a** *PHO2*, **b** *IPS1* and **c** *miR399* in Pi-starved rice seedling (cv. Nippobare) roots. The expression levels are relative to the +P condition at time zero. Relative expression levels were normalized to that of the internal control, *Os-ACTIN*

A time-course expression data for miR399, alongside data for IPS1 and PHO2 transcripts to test reproducibility against previously published data, were obtained for plants under Pi stress, using quantitative RT-PCR. The level of PHO2 mRNA shows a progressive drop, most steeply at earliest times, to about half the original level over 11 days of Pi stress (Fig. 1a); IPS1 appears to increase exponentially to extremely high levels over the same timescale (Fig. 1b). In contrast, there is a delay of at least 24 h before miR399 levels begin to increase in a roughly sigmoid manner (Fig. 1c). From these data, it seems unlikely that early decline in PHO2 mRNA levels can be directly attributed to the effects of miR399.

### A new mathematical model of the molecular regulation of phosphate uptake

**Fig. 2:**
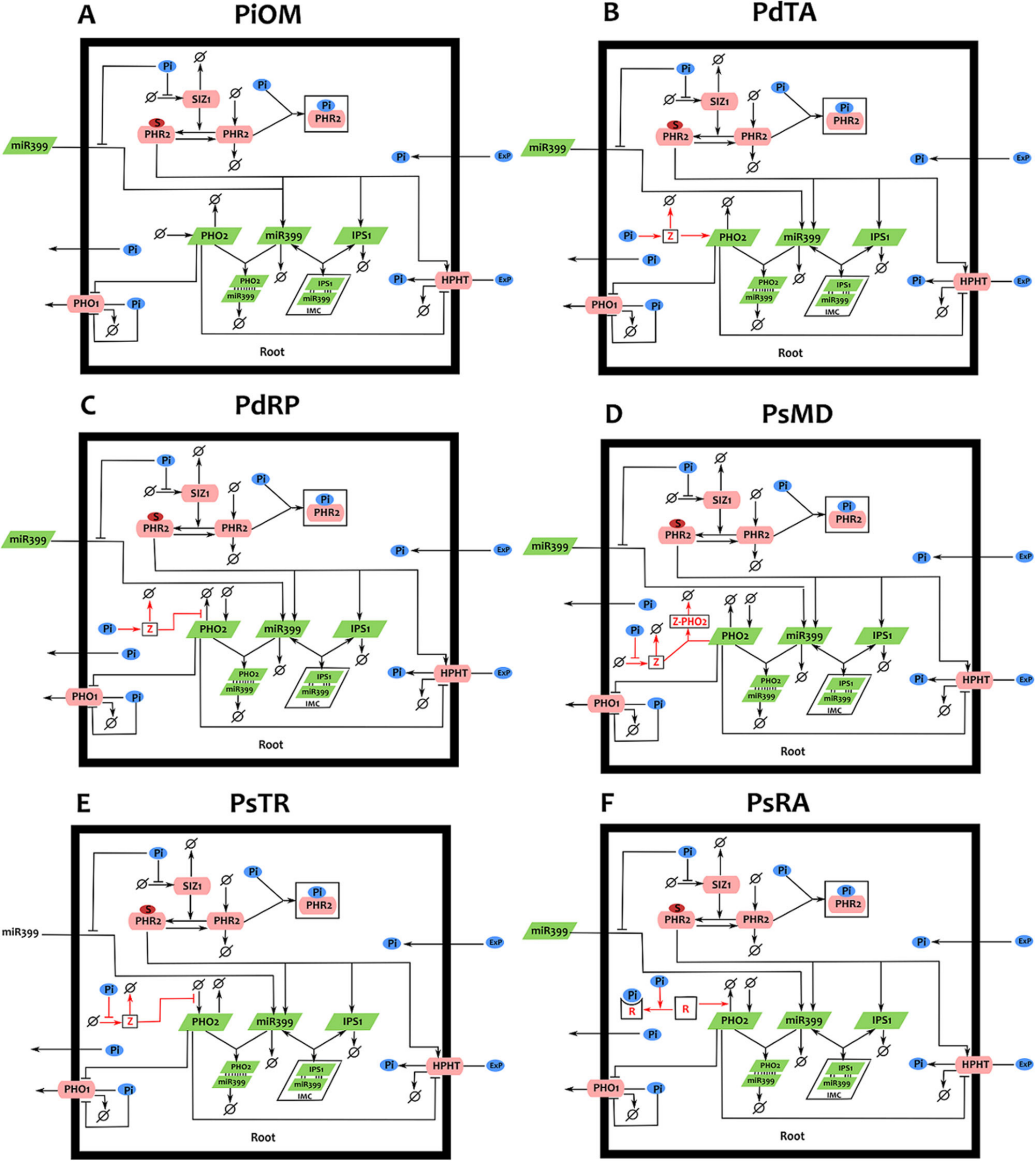
Schematic representation of the molecular regulation of Pi uptake. **a** A simplified depiction of the molecular network regulating Pi acquisition. **b**–**f** five hypotheses to explain the observed PHO2 transcript dynamics under Pi-deficient conditions. Panels **b**–**f** depict individual hypotheses (models) in a single root cell (thick black box). Red lines and letters denote the assumed reactions and species, respectively. Each hypothesis assumed the presence of an unknown regulator Z **b**–**e** or R (F) acting in different manners. Protein, ligand, mRNA, complexes and Pi ions are denoted by rectangles (pink), red ellipse, parallelogram (green), squares (transparent) and blue ellipse, respectively. Pointed solid arrows denote direct interactions and fluxes, barred arrows denote inhibition or repression, and dashed lines represent indirect interactions. ø denotes endogenous production and degradation of the molecules

The published^20,21^ and the presented (Fig. 1) experimental data pose two questions about the regulation of the PHO2 and IPS1. First, what causes the drop in PHO2 mRNA at early times upon Pi stress, given that corresponding levels of miR399 are quite low? Second, what causes the extreme elevation of IPS1 in response to Pi starvation, and its steep and sudden decline following Pi re-supply? To address these questions, a new mathematical model for the regulation of phosphate uptake in plants has been developed by adopting the molecular network presented as Fig. 2a. The model includes key regulators, both from upstream and downstream of miR399, PHO2 and IPS1: SIZ1, PHR2, PHTs and PHO1. This is primarily to explore the impact of miR399, PHO2 and IPS1 on phosphate uptake and allow the model to integrate other aspects of Pi homeostasis and other stress conditions.^22–24^

This base model, called the Pi Original Model (PiOM), is a system of ten coupled non-linear ordinary differential equations and defined in Supplementary Methods (Equation 1–10). The values of some parameters were identified from the literature, rationally assumed or calculated (see Supplementary Table 2), while some were inferred by fitting the model to the experimental data as described in the Supplementary Table 3. The available experimental data points include: fold changes in the miR399, PHO2 mRNA and IPS1 levels in response to Pi starvation presented in Fig. 2; and published mRNA-SEQ data, particularly for *PHO2*.^20,21^ Later, the base model was modified to test five competing hypotheses to explain unknown mechanisms regulating *PHO2* transcription and two potential hypotheses for IPS1 dynamics (see below).

### PiOM fits IPS1 and miR399 data but poorly predicts PHO2 dynamics

**Fig. 3:**
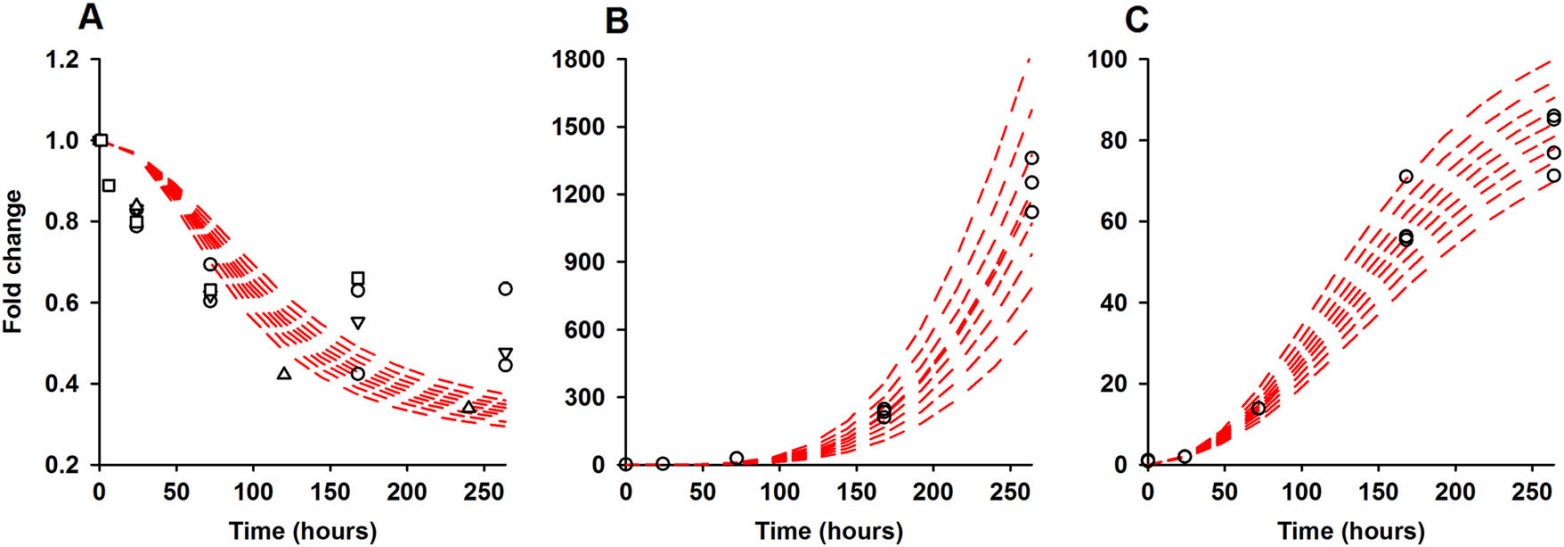
Model fitting to time-course data. Circle represents the qRT-PCR data. Square and triangles denotes fold change mRNA-seq data adopted from,^20, 21^ respectively for **a** PHO2, **b** IPS1 and **c** miR399. Prediction intervals of 80, 85, 90, 95 and 100% are shown as red dashed lines. Only three data points for PHO2 fall within the certainty limits of prediction interval of the model, unlike IPS1 and miR399 which almost all lie within the 80% interval

The PiOM fit to the experimental (fold change) data for IPS1 and miR399 is good (Fig. 3b, c), including the induction of miR399 and IPS1 and their minimal steady state expression under both Pi-deficient and sufficient conditions. The level of PHO2 mRNA starts to decline as early as 6 h following Pi stress but does not disappear completely.^20^ Figure 3a shows that the model reflects the latter, presumably because IPS1 reversibly sequesters the bulk of the miR399 available in the roots. However, the model does not correspond with the early drop in PHO2 mRNA level. This is because the level of miR399 increases in roots after 24 h of Pi stress and indicates that something else is affecting PHO2 transcript levels, particularly at early times.

### Potential hypotheses and mechanistic models for early PHO2 loss

Five hypotheses are considered as potential mechanistic explanations for the observed PHO2 transcript dynamics. These are diagrammatically presented in Fig. 2b–f and mathematically encoded as modified versions of the PiOM base model given in Supplementary Methods (Equations 11–17). The first four hypotheses assume some unknown regulator Z acting as (i) a Pi-dependent TA of *PHO2* (PdTA), (ii) a Pi-dependent protector of PHO2 mRNA (PdRP), (iii) a Pi-sensitive binder of PHO2 mRNA causing mutual degradation (PsMD), or (iv) a Pi-sensitive transcriptional repressor of *PHO2* (PsTR). The fifth hypothesis assumes a Pi-sensitive RNase promoting degradation of PHO2 mRNA in the absence of CytoPi (PsRA). Each hypothesis was tested as an individual model with an attempt to fit the complete data set while using the previously estimated parameters from the PiOM base model as initial values.

### PsTR model best explains the early regulation of PHO2 in Pi-deficient condition

**Fig. 4:**
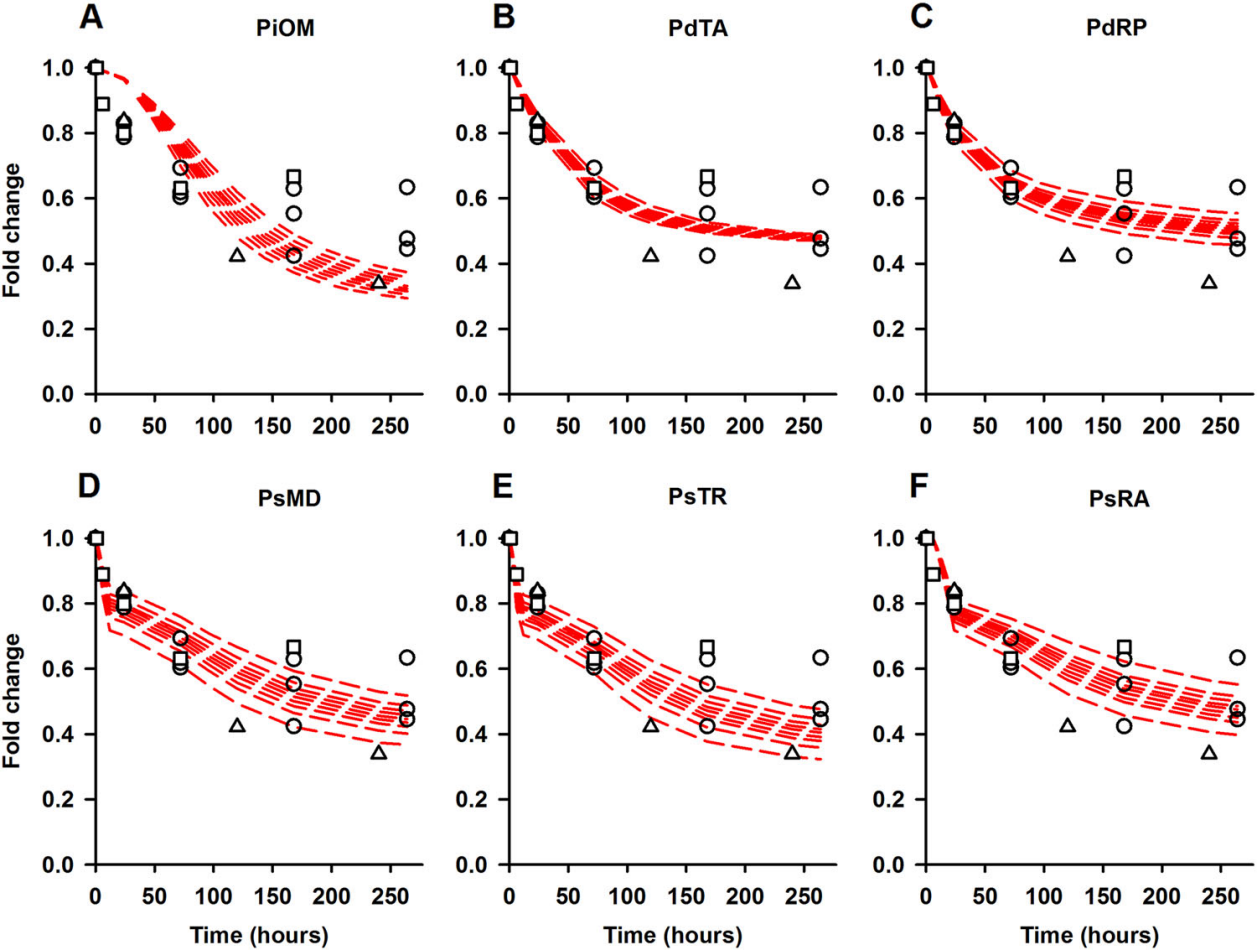
Fitting of different hypothesis models to PHO2 data. Panel **a** represents the fits of PiOM model. Panels **b**–**f** depicts the fit offered by individual hypothesis, labeled in the respective panel. Red dashed lines represent the 80% prediction interval of the respective hypothesis model. These simulations have been carried out by sampling parameter values from normal (Gaussian) distributions with means and standard deviations given from the parameter fitting in Monolix. Circle represents the qRT-PCR data. Square and triangles denotes fold change mRNA-seq data adopted from,^20, 21^ respectively

All five models show a good fit to the PHO2 (Fig. 4), IPS1 and miR399 data set in Pi-deficient conditions (see Supplementary Figs. 2 and 3) with -2 log-likelihood values lower than the original model. Using the inferred Akaike Information Criterion (AIC), the values from statistical analysis indicate that the hypothesis models offer significantly better fits to the data than the original model, see Table 1. Among the five hypotheses, PdRP gives the lowest AIC value and, statistically, it is the best hypothesis for PHO2 transcript dynamics. However, there is a qualitative difference between the models in predicting the drop in *PHO2* level at 6-h time point following Pi starvation. This feature of PHO2 is only captured by PsMD and PsTR models. Altogether, this exhibits a need to distinguish between qualitatively correct and statistically sound models, which can be achieved by experimentally determining when PHO2 mRNA levels start to decrease upon Pi stress.

**Table 1:** List of AIC, −2 log-likelihood and *P*-value for the hypothesis models

Model	AIC^a^	−2LL^a^	T^b^	△AIC^c^	k^d^	*P*-value^e^
PiOM	238.7	142.7	48	–	–	–
PdRP	192.2	88.2	52	46.5	4	4.14E-11
PdTA	206.58	106.58	50	32.12	2	1.43E-08
PsRA	211.51	111.5	50	27.19	2	1.69E-07
PsMD	218.86	106.86	56	19.84	8	1.88E-05
PsTR	234.31	122.31	56	4.39	8	8.96E-03

^**a**^ The AIC and log-likelihood (−2LL) values were generated for each model while estimating parameters using MONOLIX

^b^ total number of parameters from both structural and statistical models

^c^ AIC – AIC(of PiOM)

^d^ T – T(of PiOM)

^e^ Probability (*χ* ^2^ k > ΔAIC + 2k).^42^ *P*-values were calculated using the *χ* ^2^ distribution (CHIDIST) function in Microsoft Excel

PHO2 mRNA levels from rice roots after 0, 3, 6 and 12 h of Pi starvation were measured using qRT-PCR (Supplementary Fig. 4). The Pi-sufficient conditions showed no evidence of being regulated by the circadian clock and, if anything, increased slightly over 12 h. In contrast, almost 80% of PHO2 mRNA was lost by 3 h of Pi stress but recovered slightly at 6 and 12 h. This result again favors the PsMD and PsTR models. It is difficult to envisage biological examples of PsMD. Biochemically, a PsMD would require a Pi-binding RNA or Pi-binding proteins that are mutually degraded with a target RNA. The first of these is highly unlikely due to mutual electrostatic repulsion, while no evidence has been found for the latter. However, PsTR is more biologically credible as proteins could contain allosteric Pi-binding pockets, making this the most probable model to explain the early PHO2 transcript dynamics. Certainly, further experimental validation is needed to confirm this. The early-time qPCR profile also implies the presence of a feedback mechanism that attempts to maintain PHO2 transcript levels. For example, PHO2 might ubiquitinate and hence degrade its own activator, analogous to the maintenance of plant hormone-associated TF levels.^25^ Such a mechanism could also explain why PHO2 mRNA does not completely disappear.

### Anomalies in the predicted profiles of PHO2 and IPS1 upon Pi re-supply

**Fig. 5:**
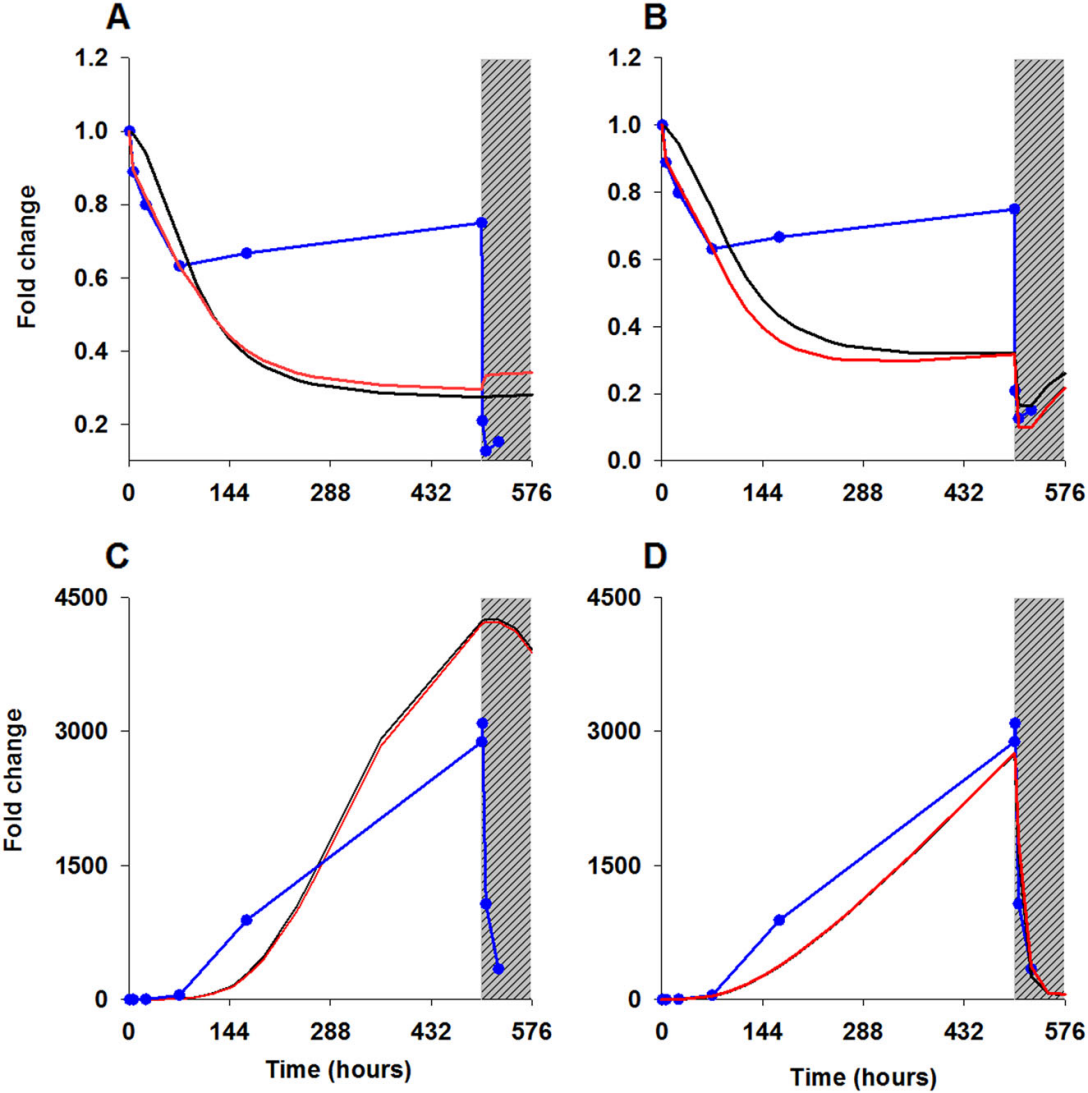
Observed and predicted profiles of PHO2 and IPS1 transcripts under Pi-depletion and repletion conditions. Panels **a** and **b** correspond to PHO2, and **c** and **d** to IPS1. Panels **a** and **c** show predicted profiles of PiOM and PsTR models, while panels **b** and **d** depict models incorporating RNA-protection. The blue dashed lines show the fold change in RNA-SEQ values from.^20^ The red and black lines denote PsTR and PiOM models, respectively. The gray zones denote the period of Pi-resupply

PHO2 and IPS1 transcript levels are observed to undergo a sudden drop within 24 h following Pi re-supply,^20^ see Fig. 5. A sudden loss of IPS1 would release the bound miR399, causing the observed rapid loss of PHO2 mRNA. Both the PiOM and PsTR models incorrectly predict the observed drastic drop in mRNA levels of PHO2 and IPS1 (Fig. 5a, c) and the plausible rise in miR399 level in response to Pi repletion (Supplementary Fig. 5a, b). This suggests some extra level of regulation concerning *PHO2* and IPS1, not represented in the current models. The fold change of *PHO2* between 200–500 h in RNAseq data is larger than those predicted by the model, Fig. 5a, b.

### Potential hypotheses for elevation of IPS1

The IPS1 level is observed to rise more than 1000 fold within 21 days of Pi stress in roots. At present, the models account for elevation of IPS1 in response to Pi deficiency by having an extremely high rate of maximal synthesis (*m*_8_ = 696) and a larger Hill co-efficient (*r* = 4) than is normally used to model transcriptional regulation;^26^ though the latter could represent an amplification mechanism. However, both models poorly predict the repletion dynamics IPS1 (Fig. 5c), showing that something is not correct. Potentially, the high IPS1 levels could arise from either a Pi-sensitive “super-transcriptional complex” causing very high rates of synthesis or a Pi-sensitive protector that impedes IPS1 degradation. These shall be considered in turn.

### Poor evidence for a super-promoter upstream of *IPS1*

Recent work on auxin-response elements^27^ show how multiple transcription factor binding sites appear to act cooperatively rather than additively to cause higher levels of induction, with seven binding sites causing the maximum stimulation of almost 30 fold over constitutive expression levels. In the case of IPS1, transcription is ~1000 times higher than constitutive levels. Around the *IPS1* promoter (see Supplementary Fig. 6), there are three copies of the P1BS motif—the binding sites for PHR2S.^28^ One of the P1BS sites is in the transcribed region, where RNA polymerase would at least temporarily separate the DNA strands, resulting in loss of PHR2S binding at that position. Furthermore, BLASTN searches with this upstream sequence revealed the presence of an unannotated tRNA-gly gene on the anti-sense strand. This makes the potential regulatory region of *IPS1* shorter (~1200 bp), and data from^20^ indicate that the tRNA gene is also expressed in response to Pi stress, see Supplementary Fig. 7. Hence, this sequence analysis does not rule out the possibility of an initiator complex that allows extremely high rates of synthesis but it seems most unlikely.

### Evidence for the protection of IPS1 RNA in Pi-deficient conditions

RNA protection has not been reported in plants but there are two lines of evidence to support this hypothesis in the case of IPS1. First, RNA stability is known to be altered by protein binding. IPS1 is predicted to have two Pumilio binding sites near its 3ʹ-end, see Supplementary Fig. 6. The role of Pumilio-RNA-binding proteins is well understood in animal systems.^29,30^ Published transcriptomic data^20^ show that the genes in this family are expressed at substantial levels and some may be elevated by Pi stress, see Supplementary Fig. 8. Another predicted protein-binding site near 3ʹ-end is for ELAV-like protein 1 (HuR). The nearest similar genes in plants are annotated as RNA-recognition and Poly-adenylate binding proteins, making these candidate IPS1-protectors as well.

The second line of evidence is from RNA structure prediction. Querying 3ʹ-end subsequence of IPS1 RNA with different sizes of the poly-A tract in BLASTN searches against NCBI-Sequence Read Archive (SRX336041) showed that the IPS1 polyA tail was up to ~18 bases (see Supplementary Fig. 13). Predicted structures of IPS1, with and without an 18-base polyA tail are shown in Supplementary Fig. 9. These indicate that the poly-A tail considerably stabilises its structure through greatly increasing double-stranded (ds) RNA formation, which would make it more resistant to attack by ribonucleases. One can envisage a protein that binds to the polyA tail (or to the polyA—polyU dsRNA region) in the absence of Pi. Upon repletion, this protein would no longer bind and IPS1 would revert to its original sensitivity to RNase degradation.

The 3ʹ-end of IPS1 also has several stretches of complementary sequences, which could potentially form a pseudoknot, see Supplementary Fig. 10. Given that an 11 base-paired RNA sequence forms a complete cycle of a double helix, the 3ʹ-end of IPS1 can form a stem-loop consisting of two hydrogen bonding regions—6 and 11 base pairs long. The terminal loop contains AATAAAG that could form the pseudoknot, which could be stablised by the polyA tail hydrogen bonding to the 5ʹ sequence to form a 12 and, potentially, 4 base-paired polyA-polyT duplexes. Much smaller pseudo-knots are known to inhibit degradation by 3ʹexonucleases in plant RNA viruses.^31, 32^ Potentially, it could play a role here. Considering everything, RNA protection is a more credible hypothesis and is worth investigating further.

### Models incorporating IPS1 RP

Using the PiOM and PsTR models, the IPS1 protection hypothesis was tested by altering the degradation rates of IPS1 and IMC as functions of CytoPi and examining Pi repletion conditions after a period of Pi stress. Thus, the magnitude of the IPS1 degradation rate $\left( {{\mathrm{d}}_7^\prime } \right)$ in the RP version of the models is altered and defined as the ratio of the original degradation rate of IPS1 (d_7_) and the steady-state initial CytoPi concentration under typical Pi condition (CytoPi, at time zero), as described in Supplementary Methods (Eqs 18–21). In these RP models, the degradation rates of IPS1 and IMC decreases in response to low Pi, allowing IPS1 to accumulate and its reversible interaction with miR399 to form more IMC complexes. However, the degradation rates of IPS1 and IMC rapidly return to normal upon Pi re-supply. The sudden degradation of IMC will thereby cause a rapid increase in the pool of miR399 and a consequent short-term decline in the PHO2 mRNA levels. PiOM-RP and PsTR-RP model were fitted to the Pi-stress data set, re-estimating the model parameters, see Supplementary Table 6.

### Models substantiate the idea of IPS1 RP in Pi-deficient conditions

Both PiOM-RP and PsTR-RP models fit the observed profiles of IPS1 and PHO2 under Pi depletion and repletion condition (Fig. 5d, b). While manually exploring the parameter space for the binding constant for IPS1-miR399 (k_7_), the value in the range of 10^−5^ s^-1^ gave the best fit and thus was adjusted accordingly to 1.9e-05, which is well still within the range of weak binding constants (i.e., 10^−5^ s^-1^) for an RNA-microRNA interaction.^33^ Notably, the re-estimated parameters for IPS1 synthesis are significantly lower, particularly the Hill coefficient “r” dropping to 2, but are in keeping with values often seen in models of gene regulation.^25,26^ These results endorse the idea of IPS1 RP under low Pi conditions and thus laboratory validation is the obvious next step. PsTR-RP is available from the BioModels database.^34^ Sensitivity and robustness analysis of all the models, with and without RP, were performed, as described in Supplementary Methods, and emphasize the very low robustness of IPS1, which presumably explains why its variable was not particularly sensitive to its Hill coefficient “r”.

## Discussion and conclusion

This work has investigated the interface between local and systemic signals in rice roots experiencing phosphate starvation, in particular the mechanisms underlying the induced early loss of PHO2 mRNA, and the extreme elevation and steep decline of IPS1 RNA, upon Pi starvation and re-supply, respectively. Reproducing the known temporal dynamics of PHO2 and IPS1 transcripts, qRT-PCR show delay in the appearance of miR399 compared to the early PHO2 mRNA loss in response to Pi starvation. This suggests the presence of an extra Pi-stress mechanism, causing an early loss in PHO2 mRNA levels.

A new mathematical model for the regulation of phosphate uptake in plants has been developed to test plausible hypotheses, concerning the regulation of PHO2 and IPS1 transcripts under different Pi conditions. Owing to the sparsity of the data, a non-linear mixed-effect modeling approach^35^ was employed for parameter estimation of the original model (PiOM). The model correctly predicts the observed temporal dynamics of miR399 and IPS1 under Pi-sufficient and deficient condition. But, the prediction for PHO2 mRNA dynamics was poor with a 24-h delay in the early drop upon Pi starvation. This can be attributed to the slow appearance of miR399, which is known and modeled as the only degrader of PHO2 mRNA. To investigate the early loss of PHO2 mRNA under Pi stress, five hypothetical mechanisms were modeled. Among these, a PsTR is the most credible hypothesis on the basis of the improved overall fit to the data, fit to the observed 6-h time point value and the prior general understanding of allosteric regulation.

To aid resolution of the hypothetical mechanisms, qRT-PCR data were generated for four time points over initial 12 h following Pi starvation. Around 80% of PHO2 mRNA is lost by 3 h, at a rate close to the normal degradation rate for RNA, and is partially restored by 6 h upon Pi stress. This is indicative of *PHO2 *regulating its own level of transcription, as the plant protein DELLA is known to regulate its own expression in a similar time scale.^25^ This leads to a hypothesis for regulation of *PHO2* expression as shown in Supplementary Fig. 11.

Sequence analysis of the upstream region 500 bp of *OsPHO2* gene predicts various potential transcription factor binding sites. This largely includes signatures for MYB, WRKY, bHLH, bZIP, MAD-box and DOF transcription factor families (see Supplementary Table 7). The expression profiles of the members from the corresponding transcription factor family under deficient Pi conditions are presented in Supplementary Fig. 12. The members in all the predicted transcription factor families show both similar and contrasting expression patterns to that of PHO2 mRNA in response to Pi starvation. However, experimental analyses are required to identity the TA of PHO2 and subsequent, characterization of its transcriptional inhibitor (TI).

In response to Pi re-supply, both PiOM and PsTR models incorrectly predict the observed steep drop in the levels of PHO2 and IPS1 RNAs, thus pointing to yet some other regulatory interaction not represented in these models. IPS1 RNA is known to sequester miR399 and accumulates to extremely high levels in plant roots during phosphate stress. Potentially, the sudden loss of IPS1 in response to Pi re-supply would release the bound miR399 causing the observed rapid loss of PHO2 mRNA. The observed IPS1 profiles can be explained by either the gene having a Pi-hypersensitive “super-promoter” synthesizing extremely high IPS1 RNA under low Pi conditions or the RNA being protected from degradation, ideally by Pi-sensitive RNA-binding proteins. Revised models incorporating RP favor the latter. Informatics analyses point to PUMILIO proteins and/or RNA secondary structure playing the role in IPS1 protection. However, exhaustive experimental investigation is required to explore this aspect of IPS1 dynamics. If confirmed, this would be the first case of regulation by RP in plant systems.

It is obviously desirable to extend systems approaches to other aspects of the phosphate-starvation response, including metabolic reprogramming. Evidence from Arabidopsis indicates that its PHR2 homolog is the key metabolic regulator,^36,37^ so the same is likely to be the case for rice. Furthermore, in rice PHR2 is known to promote the synthesis and secretion of enzymes and organic acids that promote mobilization of internal and external Pi.^38,39^ Although it would be comparatively easy to extend the model to include PHR2 regulation of metabolism and re-cycling, it extremely difficult to estimate the corresponding model parameters. At present, the only available integrated transcriptomic and metabolomics rice study considers only one time point,^40^ and hence is of little value for parameter estimation. Indeed, this aspect of plant phosphate regulation deserves more research attention.

Sensitivity analysis shows that cytosolic Pi levels are most sensitive to the internal utilization rate (U) and hence is another important aspect to study. However, this parameter covers a range of phenomena that require careful consideration, including vacuolar uptake, and incorporation into lipids and nucleic acids. The above metabolic reprogramming might be expected to reduce this utilization rate, and some vacuolar phosphate may be released back into cytosol. Furthermore, the above-mentioned exudates normally result in some extra Pi becoming available, allowing a higher rate of utilization. Inter-compartmental Pi dynamics would be fascinating to model but almost impossible to study in vivo, especially in intact plants. However, root mass continues to increase during Pi starvation.^9^ For all these reasons, this first modeling study has maintained a fixed utilization rate in order to gain a better understanding of the interactions in the local and systemic components of the starvation response.

In summary, this work has led to three hypotheses concerning the regulation of Pi uptake in rice: (1) PHO2 protein mediates the degradation of its own TA to maintain constant PHO2 mRNA levels; (2) The binding affinity of the TA of PHO2 is impaired by a phosphate-sensitive transcriptional repressor/inhibitor (TI); and (3) IPS1 RNA is protected from degradation by phosphate-sensitive RNA binding proteins. It is anticipated that this work will enable further studies and development in plant phosphate research, ultimately informing the development of crop varieties with improved Phosphorus Use Efficiencies.

## Materials and methods

### Plant materials and growth conditions

Rice (*Oryza sativa* cv. Nipponbare) was used across all the experiments. Seeds were first pre-germinated in a wet paper towel in tap water at 28 °C for 5 days before being transferred into +Pi solution. Seeds For both the initial and later experiments, the 15-days old rice seedlings were transferred into the –Pi solution for 11 days and 24 h, respectively. Hydroponic experiments were performed under controlled conditions (day/night temperature of 26/22 °C and a 12−h photoperiod, 200 µmol photons m^−2^ s^−1^) using a randomized experimental design, allowing 0.5 L of hydroponic solution per plant. The rice culture solution previously described in^20^ was used in the hydroponic growing system. For Pi-sufficient (+Pi) condition, 320 µM NaH_2_PO_4_ was used. While, NaH_2_PO_4_ was replaced by 320 µM NaCl for Pi-deficient (−Pi) solution. The pH of the solution was adjusted to 5.5, and renewed every 3 days. All experiment procedures such as media replacement and sample collection were performed at a similar time of the day to minimize possible circadian effects.

### RNA isolation and qRT-PCR analysis

Total RNA from frozen root samples, pooled from five plants, was isolated using TRIzol reagent (Life Technologies) followed by treatment with DNase I (Qiagen) and column clean-up using the RNeasy mini kit (QIAGEN) to eliminate genomic DNA contamination. cDNA was synthesized using 5 μL of total RNA (500 ng) using SuperScript III First-Strand Synthesis kit (Invitrogen, Catalog No. 18080–400). The synthesized cDNA is cleaned using an enzyme mix included in the kit (*E. coli* RNase H). qRT-PCR was performed using PerfeCTa qPCR SuperMix on a LightCycler 480 Real-Time PCR system (Roche) according to the manufacturer’s instructions. Relative expression levels were normalized to that of an internal control, *Os-ACTIN*. qRT-PCR for quantification of mature miR399 was performed following a published protocol^41^ and the corresponding primers are published in.^10^ PHO2, IPS1 and Actin primers used for qRT-PCR are listed in Supplementary Table 8. For Fig. 1, the experiments conducted with *n* (biological replicates) = 2 for IPS1 and miR399 at 0, 24, and 72 h; *n* = 4 for IPS1 and miR399 at 168 and 264 h, and *n* = 3 for PHO2. For all other qRT-PCR experiments three biological replicates were used.

### Parameter estimation

The unknown model parameters were inferred by non-linear mixed-effects models implemented in the software named MONOLIX (MOdèles NOnLInéaires à effects miXtes), version 4.33 s^35^ freely available at (http://www.lixoft.eu/). This software consists of algorithms that combine the stochastic approximation of expectation maximization algorithm with a Markov chain Monte Carlo procedure to estimate the maximum likelihood of the model parameters without any linearisation techniques. In MONOLIX, the statistical models are evaluated by using analytical model selection tools, which includes information criteria such as AIC and Bayesian information criterion, and statistical tests such as Likelihood Ratio test and Wald Test. Such evaluation tools allow the building of improved statistical models, enhancing the precision of the estimates for the parameters. More details on model fitting using MONOLIX are given in Supplementary Methods.

### Centroid secondary structure prediction of IPS1 mRNA

RNA structures were generated using RNAfold (rna.tbi.univie.ac.at) with default settings. Centroid, rather than minimum free-energy, structures were generated as these show the most probable base-pairing regions in the ensemble of secondary structures likely to be found near the most thermodynamically stable conformation.

### Data availability

The data and figures generated during this study are available in FigShare (https://figshare.com/projects/Regulatory_feedback_response_mechanisms_to_phosphate_starvation_in_rice/26098). The mathematical model is available at Biomodels with ID MODEL1702210000 (http://www.ebi.ac.uk/biomodels/).

## Electronic supplementary material

Supplementary Methods

Supplementary Tables

Supplementary Figures

## Electronic supplementary material

**Supplementary information** accompanies the paper on the *npj Systems Biology and Applications* website (10.1038/s41540-017-0041-0).
