# Supplementary material for: Regulatory feedback response mechanisms to phosphate starvation in rice
Source: NPJ Syst Biol Appl. 2018 Jan 8;4:4. doi: 10.1038/s41540-017-0041-0 (PMC5758793; doi:10.1038/s41540-017-0041-0)
Supplement: Supplementary file 1 — Supplementary Methods [file 41540_2017_41_MOESM1_ESM.pdf]

Supplementary Methods

Modelling and parameter estimation

Background:

The regulatory network of phosphate (Pi) uptake in plants includes seven key molecular members, abbreviated to SIZ1, PHR2, IPS1, miR399, PHO2/LTN1, PHO1 and PHTs, see Supplementary Information 1 (SI.1) Figure 1. SIZ1 alters the activity of proteins by covalently attaching SUMO peptides. PHR2 (equivalent to PHR1 in Arabidopsis) is a transcription factor that is activated by SIZ1 under Pi-deficient conditions and directly binds to the promoters of IPS1 - a long non-coding RNA and miR399 [1, 2]. Next, miR399 causes mutual degradation of expression of PHO2 mRNA, which encodes a ubiquitin-conjugating enzyme E24 (UBC24) that causes the degradation of PHTs (high-affinity phosphate uptake transporters) and PHO1 proteins, which move Pi into the xylem [3, 4]. In addition, IPS1 reversibly sequesters miR399 to tightly regulate PHO2 level when the Pi supply is limited [5].

A recent comprehensive time-course RNAseq experiment has offered an insight into the dynamics of rice transcriptome during the early and late stages of Pi deprivation and recovery [6]. Among these, PHO2 mRNA displayed a drop in its level at early stages of Pi starvation. On the other hand, IPS1 RNA showed drastic elevation in response to Pi starvation but undergoes sudden and steep decline upon Pi re-supply [6]. The exact mechanism underlying these features of PHO2 and IPS1 dynamics is not understood, thus revealing the need for further investigation. Mathematical modelling approach is implemented to bridge the gap in the understanding of PHO2 and IPS1 dynamics in response to Pi starvation and re-supply.

Mathematical model

A novel mathematical model describing the regulation of phosphate uptake in plants have been developed, using the molecular network presented in main text as Figure 2A. The acronyms for variable names and the corresponding initial values are defined in Supplementary Table 1.

Model assumptions

The model represents phosphate regulation in a hydroponic growth chamber, to represent the experimental set up. The root is represented as a single compartment with constant volume, flanked by external solution and vasculature. This compartment acts as a sink for external phosphate and a source for the internal plant phosphate. The decline in

compartmental phosphate levels – Cytosolic Pi (CytoPi) (in seconds and minutes) and the induced genetic/molecular response (hours and days) occur at different time-scales.

In the model, both low-affinity and high-affinity phosphate transporters are assumed to be embedded in the cell membrane, corresponding to all families of Pi transporters. The constitutively expressed low-affinity transporters account for some basal Pi uptake under all conditions. The rate of phosphate utilization is assumed to be solely dependent upon availability of Pi in cytosol. The external Pi concentration in the model is considered to remain constant under typical and deficient conditions at 200 μM and 0.0001 μM respectively. Given the upper limit for the concentration of cytoplasmic phosphate (i.e. 25mM) [7] and the proportion of it stored in the vacuoles (~80%) [8, 9], the CytoPi level is set at a steady state value of 5000 μM (i.e. 5mM) under typical hydroponic external Pi conditions, i.e. 200 μM.

The abundance of each mRNA species is used as a proxy for its protein concentration, assuming that mRNA concentration is at quasi steady state. Because the molecular regulation of Pi uptake is largely conserved between rice and *Arabidopsis* [1], the half-lives for all the transcripts in the model correspond to those of *Arabidopsis* [10]. Published and qRT-PCR results shows that PHR2 mRNA levels (and hence total protein) remain roughly constant, in both typical and low Pi conditions. PHR2 is defined as the level of unbound PHR2 available for sumoylation by SIZ1. SUMOylation of PHR2 is countered by de-SUMOylating enzymes, potentially SUMO proteases [11, 12], whose activity is assumed to be constant.

The rate of PHO2 mRNA synthesis is also assumed to be constant because there is no evidence to the contrary. The binding of IPS1-mR399 is weaker than PHO2-miR399 binding because of the 3bp insertion in the middle of miR399 binding site in IPS1, while the dissociation rate of the IPS1-miR399 (IMC) complex is assumed to be lower than their association rate.

Equations

The model is a system of ten coupled non-linear ordinary differential equations (ODEs). The corresponding model variables are: the concentration of active SIZ1 protein; the concentration of free un-sumoylated PHR2 transcription factor; the concentration of sumoylated PHR2 transcription factor; the concentration of miR399 RNA; the concentration of PHO2 mRNA; the concentration of PHO1 protein; the concentration of membrane-bound high-affinity PHT protein; the concentration of IPS1 RNA; the concentration of IPS1-miR399 complex (IMC); and the concentration of CytoPi in roots.

The rate of endogenous production and degradation are respectively denoted by “m” and “d”, along with relevant subscripts and square brackets denote concentrations. The equations are presented below.

The active SIZ1 protein is repressed by cytosolic phosphate (CytoPi) with the inhibition constant k_1 and Hill co-efficient n_1 (Equation 1). Under typical Pi conditions, the transcription factor PHR2 remains bound to Pi-stabilised SPX4 protein [13]. For simplicity, PHR2 is assumed to interact directly with CytoPi (Equation 2). The free unbound PHR2 is sumoylated with Michealis constant k_3 and eventually, de-sumoylation with rate constant k_4 (Equation 3). The bulk translocation of miR399 from shoot-to-root (β) is repressed by CytoPi. PHR2S triggers the production of miR399 with activation constant k_5 and Hill co-efficient n (Equation 4). miR399 binds with constitutively expressed PHO2 with rate constant k6, causing their mutual degradation (Equation 5). The xylem-loading proteins PHO1 are suppressed by CytoPi and PHO2 (Equation 6). PHR2S triggers the production of PHT proteins with activation constant k_9 and Hill co-efficient n and are repressed by PHO2 (Equation 7). PHR2S elicits the production of IPS1 with the activation constant k_{10} and Hill co-efficient r. IPS1 sequesters miR399 to form reversible complex – IMC (Equation 8 and 9). PHT proteins and constitutive low-affinity transporter proteins take up phosphate (E) from a constant external source with their respective Vmax and Km. The acquired Pi is loaded into the xylem via PHO1 proteins following Michealis-Menten kinetics. Depending on the availability, CytoPi is stored or utilized in the compartment with the rate constant (U).

PiOM model equations:

$$\frac{d[SIZ1]}{dt} = \frac{m_1\, k_1^n}{k_1^n + [CytoPi]^n} - d_1[SIZ1] \tag{1}$$

$$\frac{d[PHR2]}{dt} = m_2 - k_2[PHR2][CytoPi] - \frac{m_3[PHR2][SIZ1]}{k_3 + [PHR2]} + k_4[PHR2S] - d_2[PHR2] \tag{2}$$

$$\frac{d[PHR2S]}{dt} = \frac{m_3[PHR2][SIZ1]}{k_3 + [PHR2]} - k_4[PHR2S] \tag{3}$$

$$\frac{d[miR399]}{dt} = \frac{\beta}{1 + [CytoPi]} + \frac{m_4[PHR2S]^n}{k_5^n + [PHR2S]^n} - k_6[miR399][PHO2] - k_7[miR399][IPS1] + k_8[IMC] - d_3[miR399] \tag{4}$$

$$\frac{d[PHO2]}{dt} = m_5 - k_6[miR399][PHO2] - d_4[PHO2] \tag{5}$$

$$\frac{d[PHO1]}{dt} = \frac{m_6}{(1 + [CytoPi])(1 + [PHO2])} - d_5[PHO1] \tag{6}$$

$$\frac{d[PHT]}{dt} = \frac{m_7[PHR2S]^n}{(k_9^n + [PHR2S]^n)(1 + [PHO2])} - d_6[PHT] \tag{7}$$

$$\frac{d[IPS1]}{dt} = \frac{m_8[PHR2S]^r}{k_{10}^r + [PHR2S]^r} - k_7[miR399][IPS1] + k_8[IMC] - d_7[IPS1] \tag{8}$$

$$\frac{d[IMC]}{dt} = k_7[miR399][IPS1] - k_8[IMC] - d_7[IMC] \tag{9}$$

$$\frac{d[CytoPi]}{dt} = \frac{k_{11}[PHT][E]}{k_{12} + [E]} + \frac{k_{13}[E]}{k_{14} + [E]} - \frac{k_{15}[PHO1][CytoPi]}{k_{16} + [CytoPi]} - U[CytoPi] \tag{10}$$

Model parameterization

The model consists of 35 parameters, namely m_i, k_j, d_l, β , U, n, and r, where i = 1, ..., 8, j = 1, ...16, and l = 1, .., 7 (see Supplementary Tables 2 and 3). The magnitude of these parameters were either adopted from the literature, assumed, calculated or estimated.

Known, assumed and calculated parameters

Values of 9 parameters, mainly the degradation rates, were obtained directly from the literature [10]. The binding constant for miR399 and PHO2 (k_6) is assumed to equal 0.009, on the basis of the work published by [14]. The Hill co-efficient (n) was set to 2 in keeping with many models of gene regulation while Hill co-efficient for IPS1 (r) was set to 4 to achieve the observed delayed and steep increase in IPS1 levels. The inhibition co-efficient for SIZ1 (k_1), PHO1 and PHT were set to unity, in order to optimize the number of parameters for estimation. The value of m_5, k_{13} and U were calculated as following, using the steady state levels of the respective variables.

Calculation for the production rate of PHO2 (m_5)

Equation for PHO2:

$$\frac{d[PHO2]}{dt} = m_5 - k_6[miR399][PHO2] - d_4[PHO2]$$

Assuming PHO2 at quasi steady state:

$$m_5 - k_6[miR399][PHO2] - d_4[PHO2] = 0$$

Under sufficient Pi conditions, $[miR399] = 0$ and $[PHO2] = 1$

Rearranging LHS:

$m5 - d_4 = 0$ i.e. $m5 = d_4 = 0.3741$

Calculation for the V_{max} for phosphate uptake by LPHT (k_{13}) and Rate constant for internal utilisation of CytoPi (U)

Equation for CytoPi:

$$\frac{d[CytoPi]}{dt} = \frac{k_{11}[PHT][E]}{k_{12} + [E]} + \frac{k_{13}[E]}{k_{14} + [E]} - \frac{k_{15}[PHO1][CytoPi]}{k_{16} + [CytoPi]} - U[CytoPi]$$

Under sufficient Pi conditions, the value of high affinity transporter [PHT] = 0 and Pi-efflux transporter [$PHO1$] = 0.

Assuming CytoPi at a quasi-steady state:

$$\frac{k_{13}[E]}{k_{14} + [E]} - U[CytoPi] = 0$$

The term for Pi uptake corresponds to the total Pi brought into the cell under normal conditions and U corresponds to the amount of Pi used or stored in vacuoles. Assuming that 80% of Pi is stored in the vacuoles, then the cytosolic Pi (CytoPi) reflects remaining 20% of the Pi brought in the cell. Thus, for the given [$CytoPi$] = 5000μM, then the cytoplasmic Pi is 25000μM, corresponding to the total Pi taken up by low affinity transporters.

$$\frac{k_{13}[E]}{k_{14} + [E]} = 25000 \qquad and \qquad U[CytoPi] = 25000$$

The values for k_{14} and E are 177 and 200, respectively.

$$k_{13} = \frac{25000}{0.5305} = 47125.353 \qquad and \qquad U = 5$$

Model fitting to data

The values of remaining 20 parameters were estimated by fitting the model to the 50 data points corresponding to the fold changes in the miR399, PHO2 and IPS1 level in response to Pi starvation. The dataset includes both generated qRT-PCR and published RNA-SEQ

data points [6]. As fold change datasets are used, no units were assigned to the parameters, except for the time in hours (h). The initial values of the variable were set to zero, with the exception of PHO2 and CytoPi, which were initially set to 1 and 5000, respectively. Model fitting was conducted using a software named MONOLIX that incorporates the suite of parameter inference techniques, detailed in the following section.

Parameter estimation using MONOLIX

Theory

The qRT-PCR data correspond to overall values for a population of multiple root cell types for 3 plants per biological replicate. With biological replication, observations are collection of n functions $f_i(t)$, i = 1...n, one for each replicate. Often, the mean population function μ(t) characterises the overall temporal behavior of the population. Furthermore, the observations are always subject to some level of measurement errors, inter-individual variability and some unexplainable variation.

Integrating the variability of these is important to understand the population behavior and can be captured using mixed-effect models. These statistical, or inference, methods simultaneously account for both fixed and random effects on the observation from small sample sizes and sparse datasets. The fixed effects are population parameters (characteristic measures of the entire population) assumed to be the same at each time the data are collected, and random effects are random variables associated with each individual sample from a population. In contrast to linear models, non-linear mixed effect models consider the mathematical model (statistically referred to as a structural model) as the non-linear function of both fixed and random effects and involves approximation during the estimation.

With this notion, the unknown (population) parameters of the mathematical model were inferred by non-linear mixed-effects models implemented in the software named MONOLIX (MOdèles NOn LInéaires à effets miXtes), version 4.33s, freely available at (http://www.lixoft.eu/). This software consists of algorithms that combine the Stochastic Approximation of Expectation Maximization (SAEM) algorithm with a Markov chain Monte Carlo (MCMC) procedure to estimate the maximum likelihood of the model parameters without any linearisation techniques. In MONOLIX, the statistical models are evaluated by using analytical model-selection tools, which includes information criteria such as Akaike information criterion (AIC) and Bayesian information criterion (BIC), and statistical tests such as Likelihood Ratio test and Wald Test. Such evaluation tools allow the building of improved statistical models, enhancing the precision of the estimates for the population parameters.

Algorithm

Initially, the individual parameters were simulated using an MCMC approach in the expectation step of SAEM algorithm. These individual parameters were then used to compute a stochastic approximation of the conditional expectation of the log-likelihood of the complete data. Subsequently, the complete log-likelihood was maximized to obtain the updated estimates of the population parameters. In practice, SAEM generates 1 to 5 random samples per individual per iteration to allow for an efficient and rapid convergence toward the solution. This "burn-in" period typically requires 200 iterations. Thereafter, the program accumulates random sample results for the next iterations, to estimate the population means and inter-individual variances.

Execution

Model adjustment

Given that IPS1 RNA is partially complementary to miR399 (with 3 base mismatch), the binding affinity of miR399 toward IPS1 would be relatively weaker than that to PHO2. However, the release of IPS1 from the IPS1-miR399 complex would be slower than its binding. Considering such a relationship, values of the parameter k_7 (the binding constant for IPS1 and miR399) and k_8 (Dissociation constant for IPS1-miR399 complex) were indirectly deduced as following:

$$k_7 = k_6/(1 + Y_1) \; ; \; k_8 = k_7 . Y_2$$

The above algebraic equation were incorporated in the model fitting to first estimate Y_1 and Y_2, which were then further used to calculate the value for k_7 and k_8. These parameters (i.e. Y_1 and Y_2) were purely included to aid the estimation and are related to the known parameter k_6 (Binding constant for miR399 and PHO2).

MONOLIX settings

The structural model (i.e. the Pi-regulation model) encoded in MLXTRAN format in a text file (Laveille, 2014). This model and the datasets were loaded onto the MONOLIX platform. Screen shot of the MONOLIX GUI platform is presented in SI.1.Figure 13. The GUI platform is divided into four frames: the data and model, the initialization, the algorithms and the results. Each of these frames requires some input for the precise estimation of parameters. In this estimation exercise following settings were used. In the data and model frame, the 'Combine1' observation model was selected for the model variable to which data are fitted. In the initialization frame, the initial value and standard deviation for the random effect for each parameter were entered. In the algorithm frame,

the default Simulated Annealing option was unselected, the number of iteration (K1 and K2) and chains were respectively set to 800, 200 and 50. A new seed was generated for every run, and for other options the default settings were used. In the result frame, linearization was selected for standard errors and log-likelihood, while the conditional mode was selected for individual parameter options. Finally, estimation can be started using the run button given in the top center. Details underlying all these options are well defined and explained in the tutorial available on the MONOLIX webpage (lixoft.eu/products/monolix/documentation).

Estimation settings

Due to the sparsity of dataset for parameters to be estimated, the inference was done by multiple sequential runs as follows. In the first run, the initial values of the parameters and their corresponding standard deviations were set to unity, and the program was executed. The resulting estimates were than assigned as the initial values for the second run. This cycle was repeated until the best fits were achieved. The algorithm initially struggled to find the range for certain parameters that would result in an acceptable fit to the extremely high IPS1 levels. Using the observation while manually exploring the parameter space, the initial values for m_8 and k_{10} were manually set to 1000 and 70 with standard deviation of unity. These adjustments gave appropriate estimates with acceptable fits to the observed data. The list for the initial values and estimates for the parameters and corresponding standard deviation from each run is given in Supplementary. Table 4.

Estimated parameters for PiOM

The estimated mean values of the all parameters their corresponding standard deviation are listed in Supplementary.Table 3. Most estimated values are within the biologically relevant range, except for the parameters concerning IPS1 synthesis, m_8 and r, whose magnitudes are unrealistically high relative to other parameters. Given the known short half-life of IPS1 (i.e ~ 5 hours), a high maximal expression level of the promoter (i.e. m_8 = 693.33) is essential to achieve the observed IPS1 level under Pi deficient conditions. The values for the rate of PHO1 production (m_6), uptake velocity for high affinity transporters (k_{11}) and kinetic parameters for CytoPi efflux by PHO1 (k_{15} and k_{16}), are relatively very small. This is due to lack of data for their respective variables. Perhaps, for a similar reason, the values for k_2 and k_4 were poorly estimated with higher standard deviation than their mean, but due to their small magnitudes they do not affect the model simulations.

Hypotheses models to explain early PHO2 dynamics in response to Pi-stress

Given the poor prediction of PHO2 profile by PiOM model, five hypotheses are considered as potential mechanistic explanations for the observed PHO2 dynamics in response to Pi-starvation. The first four hypotheses assume some unknown regulator Z acting as (i) a Pi-dependent transcriptional activator of PHO2 (PdTA) - eqs. 11 and 12, (ii) a Pi-dependent protector of PHO2 mRNA (PdRP) - eqs. 11 and 13, (iii) a Pi-sensitive binder of PHO2 mRNA causing mutual degradation (PsMD) - eqs. 14 and 15, or (iv) a Pi-sensitive transcriptional repressor of PHO2 (PsTR) - eqs. 14 and 16. The fifth hypothesis assumes a Pi-sensitive RNase promoting degradation of PHO2 mRNA in absence of CytoPi (PsRA) - eq. 17. All these hypotheses are diagrammatically presented in main text as Figure 2. Each hypothesis was tested as an individual model with an attempt to fit the complete data set. In this round of parameter estimation, previously estimated parameters in Supplementary Table 3, were used as the initial values. The parameter estimates for each hypothesis model is listed in Supplementary Table 5.

PHO2 hypotheses equations

$$\frac{d[Z]}{dt} = m_9[CytoPi] - d_8[Z] \tag{11}$$

$$\frac{d[PHO2]}{dt} = m_5(1 + [Z]) - k_6[miR399][PHO2] - d_4[PHO2] \tag{12}$$

$$\frac{d[PHO2]}{dt} = m_5 - k_6[miR399][PHO2] - \frac{d_4[PHO2]k_{17}}{k_{17} + [Z]} \tag{13}$$

$$\frac{d[Z]}{dt} = \frac{m_9 k_{18}}{k_{18} + [CytoPi]} - d_8[Z] \tag{14}$$

$$\frac{d[PHO2]}{dt} = m_5 - k_6[miR399][PHO2] - d_4[PHO2] - k_{19}[PHO2][Z] \tag{15}$$

$$\frac{d[PHO2]}{dt} = \frac{m_5 k_{19}}{k_{19} + [Z]} - k_6[miR399][PHO2] - d_4[PHO2] \tag{16}$$

$$\frac{d[PHO2]}{dt} = m_5 - k_6[miR399][PHO2] - d_4[PHO2] - \frac{k_{20}[PHO2]}{1 + [CytoPi]} \tag{17}$$

IPS1 protection hypothesis model

Two strike features concerning the dynamics of IPS1 have been observed. The first is its extreme and unremitting elevation following 21 days of Pi stress and the second is its sudden drop within 24 hours of Pi repletion. The exact reason underlying these features of IPS1 dynamics is vaguely understood. Though the model simulation is somewhat able to explain IPS1 and PHO2 dynamics in response to Pi-starvation. However, the simulation incorrectly predicts the observed drastic drop in the IPS1 and PHO2 level in response to Pi re-supply. This suggests that there must be some extra level of regulation concerning IPS1 and PHO2, which is not correctly represented in the current models.

Compared to other, RNA protection hypothesis was identified as the most plausible explanation for the observed elevation in the level of IPS1 in response to Pi-stress and its sudden drop upon Pi re-supply. Using the PiOM and PsTR models, the IPS1 protection hypothesis was tested by modulating the degradation rate of IPS1 and IMC as the function of CytoPi and examining Pi repletion conditions after a period of phosphate stress. Furthermore, the magnitude of the IPS1 degradation rate in the RNA Protection (RP) version of the models was altered (d_7') and is defined as the quotient of original degradation rate of IPS1 (d7) and the steady-state initial CytoPi concentration under normal Pi condition (CytoPi, at time = 0), see equations (18 - 21). In these new models, the degradation rates of IPS1 and IMC will decrease in response to low Pi, allowing IPS1 to accumulate and its reversible interaction with miR399 to form more IMC complexes. However, the degradation rates of IPS1 and IMC rapidly return back to normal upon Pi-resupply. The sudden degradation of IMC will thereby cause a rapid increase in the pool of miR399 and consequent short-term decline in PHO2.

PiOM-RP and PsTR-RP model were fitted to the Pi-stress data set and parameters were re-estimated. In this round of parameter estimation, previously estimated parameters in Supplementary Tables 3 (for PiOM) and 5 (PsTR), were used as the initial values. The parameter estimates and corresponding standard deviation for the RP models are listed in Supplementary Table 6. The new values for the rate of IPS1 synthesis and corresponding Hill coefficients are lower for both PiOM and PsTR model and are more consistent with values typically seen in models of gene regulation. The models can be fitted to the mRNA-SEQ PHO2 profile by either increasing the value of IPS1- miR399 binding constant (k_7) to 10^{-4} or setting IPS1 production rate (m_8) to 200. However, the biological relevance of these values is uncertain.

$$\frac{d[miR399]}{dt} = \frac{\beta}{1 + [CytoPi]} + \frac{m_4[PHR2S]^n}{k_5^n + [PHR2S]^n} - k_6[miR399][PHO2] - k_7[miR399][IPS1] + k_8[IMC] - d_3[miR399] + d_7'[IMC][CytoPi] \tag{18}$$

$$\frac{d[IPS1]}{dt} = \frac{m_8[PHR2S]^r}{k_{10}^r + [PHR2S]^r} - k_7[miR399][IPS1] + k_8[IMC] - d_7'[CytoPi]([IPS1] - [IMC]) \tag{19}$$

$$\frac{d[IMC]}{dt} = k_7[miR399][IPS1] - k_8[IMC] - d_7'[CytoPi][IMC] \tag{20}$$

$$d_7' = \frac{d_7}{CytoPi_{time-0}} = \frac{0.1386}{5000} = 2.7e - 05 \tag{21}$$

Sensitivity and Robustness analysis

Global sensitivity analysis was performed to investigate non-linear effects of the model parameters on the model output by simultaneously varying all of them. All 12 models, with and without RNA protection, were encoded in SBML format [15] using COPASI [16] and uploaded into an open-source software tool named, Systems Biology Markup Language based Sensitivity Analysis Tool (SBML-SAT) to perform sensitivity and robustness analysis [17]. Under default settings, the multi-parametric algorithm was used to perform global-sensitivity analysis. This algorithm randomly generates parameter values from a pre-defined uniform probability distribution, thereby mapping the uncertainty of the parameters into the model output. The obtained sensitivities from the integrated response correspond to perturbations in each model variable (i.e. the area under the curve) with respect to the variation in the parameter during the time-course. Sensitivity analysis was conducted for all the models, with and without RNA protection. As no significance difference was observed in the sensitivities of the variable between different models (data not shown), only the sensitivity heat-map of PiOM model is presented here, as Figure SI3.1.

The integrated response (i.e. total sensitivity of the model output across the time-course with respect to the variation of parameters) showed, as expected, that most variables were mainly sensitive to their synthesis and degradation rates. However, most variables (most especially SUMOylated-PHR2) are sensitive to the parameter (m2) - the rate of PHR2 production, Figure S1. As PHR2 is central to the regulation of the phosphate-starvation response, its rate of synthesis is bound to affect various downstream variables. The concentration of CytoPi is most sensitive to the rate of its internal utilisation (U), which had no effect on any of the other variables.

Robustness is the property of a system that maintains its function(s) under perturbations. Robustness analysis was performed to investigate the robustness of the model variables against the total parameter variation. The Robustness-coefficient identified from this analysis was recorded for all the variables from respective output plots and were replotted together as the bar-chart, Figure S2. For more details about this analysis can be found in [17]. This analysis identifies CytoPi as the most robust variable underlining the fact that the system always tries to keep its concentration constant. The high-affinity phosphate transporters (PHTs) are the next most robust variable, reflecting system's need to use these transporters only when necessary. On the other hand, IPS1 is the least robust

variable, reflecting its very large change in concentration. Interestingly, IPS1 become relatively more robust upon the inclusion of RNA protection in the models. However, there is no or negligible effect of RNA protection on the robustness of other variables. It also worth noting that IPS1 shows a large variation in its level between different rice varieties upon Pi stress [18].

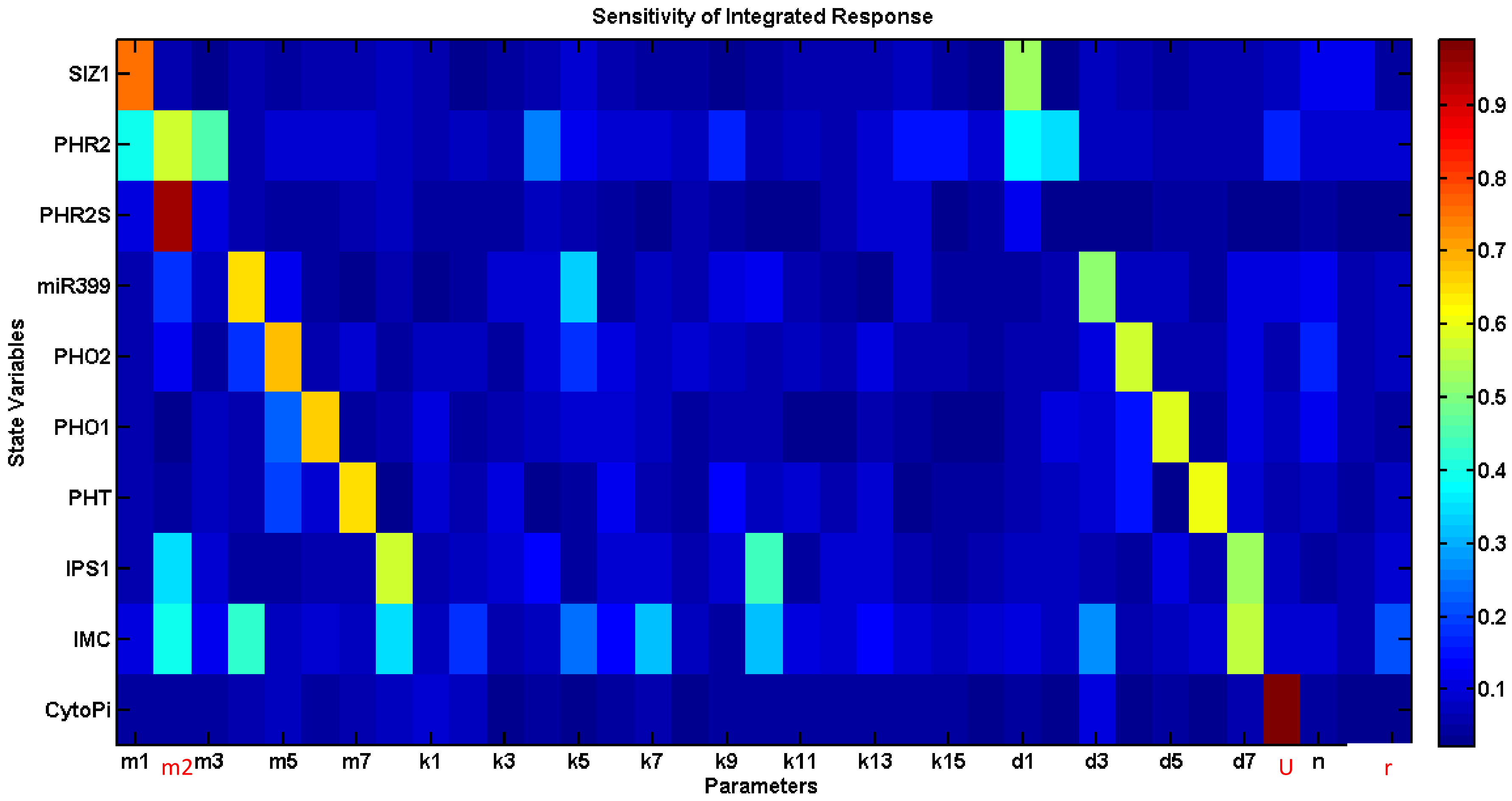

Figure S1: Global sensitivity analysis of the PiOM model. The heat-map depicts the multi-parametric global sensitivities for the integrated response of different parameters on the system variables. The sensitivities are between 0 and 1, where a value closer to 1 reflects the higher sensitivity of the model output to the respective parameter. The parameters m2 and U have the most pronounced effects upon variables, and are shown in red.

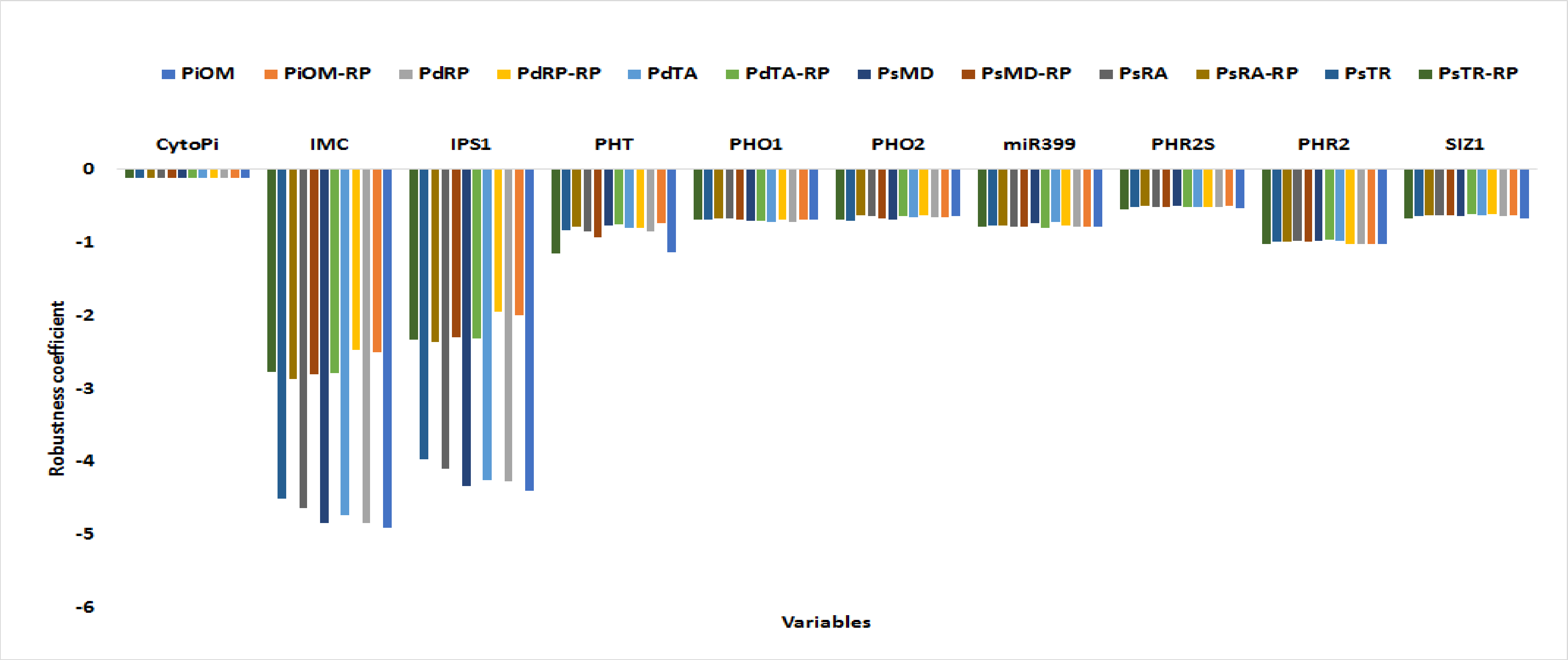

Figure S2: Robustness analysis of the parametrised model. The plot presents the robustness of the individual variables in the model. The robustness co-efficient (R) is usually calculated as a negative value. The closer the R to zero, the more robust the variable to perturbation (parameter variation).

References:

1. Chiou, T.J. and S.I. Lin, *Signaling network in sensing phosphate availability in plants.* Annu Rev Plant Biol, 2011. **62**: p. 185-206.
2. Lin, W.Y., S.I. Lin, and T.J. Chiou, *Molecular regulators of phosphate homeostasis in plants.* J Exp Bot, 2009. **60**(5): p. 1427-1438.
3. Bari, R., et al., *PHO2, microRNA399, and PHR1 define a phosphate-signaling pathway in plants.* Plant Physiol, 2006. **141**(3): p. 988-999.
4. Hu, B., et al., *LEAF TIP NECROSIS1 plays a pivotal role in the regulation of multiple phosphate starvation responses in rice.* Plant Physiol, 2011. **156**(3): p. 1101-1115.
5. Franco-Zorrilla, J.M., et al., *Target mimicry provides a new mechanism for regulation of microRNA activity.* Nat Genet, 2007. **39**(8): p. 1033-1037.
6. Secco, D., et al., *Spatio-Temporal Transcript Profiling of Rice Roots and Shoots in Response to Phosphate Starvation and Recovery.* Plant Cell, 2013. **25**(11): p. 4285-4304.
7. Schachtman, D.P., R.J. Reid, and S.M. Ayling, *Phosphorus Uptake by Plants: From Soil to Cell.* Plant Physiology, 1998. **116**(2): p. 447-453.
8. Bieleski, R.L., *Phosphate Pools, Phosphate Transport, and Phosphate Availability.* Annual Review of Plant Physiology, 1973. **24**(1): p. 225-252.
9. Poirier, Y. and M. Bucher, *Phosphate transport and homeostasis in Arabidopsis.* Arabidopsis Book, 2002. **1**: p. e0024.
10. Narsai, R., et al., *Genome-wide analysis of mRNA decay rates and their determinants in Arabidopsis thaliana.* Plant Cell, 2007. **19**(11): p. 3418-3436.
11. Conti, L., et al., *Small ubiquitin-like modifier proteases OVERLY TOLERANT TO SALT1 and -2 regulate salt stress responses in Arabidopsis.* Plant Cell, 2008. **20**(10): p. 2894-908.
12. Kurepa, J., et al., *The small ubiquitin-like modifier (SUMO) protein modification system in Arabidopsis. Accumulation of SUMO1 and -2 conjugates is increased by stress.* J Biol Chem, 2003. **278**(9): p. 6862-72.
13. Lv, Q., et al., *SPX4 Negatively Regulates Phosphate Signaling and Homeostasis through Its Interaction with PHR2 in Rice.* Plant Cell, 2014. **26**(4): p. 1586-1597.
14. Zinovyev, A., et al., *Mathematical modeling of microRNA-mediated mechanisms of translation repression.* Adv Exp Med Biol, 2013. **774**: p. 189-224.
15. Hucka, M., et al., *The systems biology markup language (SBML): a medium for representation and exchange of biochemical network models.* Bioinformatics, 2003.19(4), 524–531.

16. Hoops, S., et al., *COPASI-a COmplex PAthway SImulator*. Bioinformatics, 2006. 22(24), 3067–3074.

17. Zi, Z., et al, S*BML-SAT: a systems biology markup language (SBML) based sensitivity analysis tool.* BMC Bioinformatics, 2008. 9, 342.

18. Oono, Y., et al., *Diversity in the complexity of phosphate starvation transcriptomes among rice cultivars based on RNA-Seq profiles*. Plant Mol Biol, 2013. 83(6), 523–537.
