# Supplementary material for: Regulatory feedback response mechanisms to phosphate starvation in rice
Source: NPJ Syst Biol Appl. 2018 Jan 8;4:4. doi: 10.1038/s41540-017-0041-0 (PMC5758793; doi:10.1038/s41540-017-0041-0)
Supplement: Supplementary file 2 — Supplementary Tables [file 41540_2017_41_MOESM2_ESM.pdf]

**Supplementary Table 1: List of model variables and their initial values.**				
**Variable acronym**	**Variable name**	**Variable type**	**Initial value**	**units**
SIZ1	SUMO E3 ligase 1	Protein	0	Fold
PHR2	Phosphate Starvation Response 2	Protein	0	Fold
PHR2S	SUMO-bound PHR2	Protein-ligand complex	0	Fold
miR399	miRNA-399	microRNA	0	Fold
PHO2	Phosphate 2	mRNA	1	Fold
PHO1	Phosphate 1	membrane protein	0	Fold
PHT	Phosphate Transporter (High-affinity)	membrane protein	0	Fold
IPS1	Induced by Phosphate Starvation	Long non-coding RNA	0	Fold
IMC	IPS1-miR399 complex	RNA-miRNA complex	0	Fold
CytoPi	Cytosolic Phosphate	Ion	5000	µM
E	External Phosphate	Ion	200	µM

Supplementary Table 2: List of known, assumed and calculated parameters.				
**Parameter name**	**Description**	**Value**	**Unit**	**Reference**
m5	Maximal production rate of PHO2	0.3742	h^{-1}	C
k1	Inhibition co-efficient for SIZ1	1	h^{-1}	A
k6	Binding constant for miR399 and PHO2	0.009	h^{-1}	R**
k12	km for phosphate uptake by HPHT	23	μM	R*
k13	Vmax for phosphate uptake by LPHT	47125	h^{-1}	C
k14	km for phosphate uptake by LPHT	177	μM	R*
d1	Rate of SIZ1 degradation	0.0289	h^{-1}	R
d2	Rate of PHR2 degradation	0.0289	h^{-1}	R
d3	Rate of miR399 degradation	0.0578	h^{-1}	A*
d4	Rate of PHO2 degradation	0.3741	h^{-1}	R
d5	Rate of PHO1 degradation	0.0866	h^{-1}	R
d6	Rate of PHT degradation	0.0866	h^{-1}	R
d7	Rate of IPS1 degradation	0.1386	h^{-1}	R'
U	Rate constant for internal utilisation of Pi	5	h^{-1}	C
n	Hill coefficient	2	*dl*	A
r	Hill coefficient for IPS1	4	*dl*	A

Abbreviations denotes following - dl: dimensionless; h: hours; PHT/HPHT: High-affinity Phosphate Transporters, LPHT: Low-affinity Phosphate Transporters; A: assumed value; A*: assumed based on information from (Meng et al., 2011, Ruegger and Grosshans, 2012); C: calculated value (for details see SI.3); R: degradation rates were calculated using the half-life values of the respective orthologs in Arabidopsis from (Narsai et al., 2007); R' : degradation rate dervied using ecovery data from (Secco et al., 2013) ; R*: kinetic value of transporters from (Nussaume et al., 2011) and R**: miRNA binding affinity from (Zinovyev et al., 2013) ; The Hill coefficients have been set greater than one to reflect a non-linear response of protein/RNA to activators and repressors.

References:

Narsai, R., et al., Genome-wide analysis of mRNA decay rates and their determinants in Arabidopsis thaliana. Plant Cell, 2007. 19(11): p. 3418-3436.

Nussaume, L., et al., Phosphate Import in Plants: Focus on the PHT1 Transporters. Front Plant Sci, 2011. 2: p. 83.

Meng, Y., et al., The regulatory activities of plant microRNAs: a more dynamic perspective. Plant Physiol, 2011. 157(4): p. 1583-95.

Ruegger, S. and H. Grosshans, MicroRNA turnover: when, how, and why. Trends Biochem Sci, 2012. 37(10): p. 436-46.

Secco, D., et al., Spatio-Temporal Transcript Profiling of Rice Roots and Shoots in Response to Phosphate Starvation and Recovery. Plant Cell, 2013. 25(11): p. 4285-4304.

Zinovyev, A., et al., Mathematical modeling of microRNA-mediated mechanisms of translation repression. Adv Exp Med Biol, 2013. 774: p. 189-224.

Supplementary Table 3: Estimated parameters and with their respective standard deviation.

Parameter name	Description	Value	s.d.	Unit
m1	Maximal production rate of SIZ1	0.422	0.004	h^{-1}
m2	Maximal production rate of PHR2	0.2	0.001	h^{-1}
m3	Vmax for PHR2 sumoylation	3.532	0.005	h^{-1}
m4	Maximal production rate of miR399	6.639	0.021	h^{-1}
m6	Maximal production rate of PHO1	0.003	0.0004	h^{-1}
m7	Maximal production rate of PHT	2.6083	0.0171	h^{-1}
m8	Maximal production rate of IPS1	693.33	0.0032	h^{-1}
k2	Rate constant for PHR2-CytoPi interaction	0.002	0.013	h^{-1}
k3	Michaelis constant for PHR2 sumoylation	1.147	0.003	h^{-1}
k4	Rate constant for PHR2S deSUMOylation	0.008	0.065	h^{-1}
k5	Activation coefficient for miR399 production	28.89	0.0006	h^{-1}
k7	Binding constant for IPS1 and miR399	2.49E-07	**	h^{-1}
k8	Dissociation constant for IMC	1.19E-07	**	h^{-1}
k9	Activation coefficient for PHT	0.769	0.017	h^{-1}
k10	Activation coefficient for IPS	64.69	0.0074	h^{-1}
k11	Partial Vmax for HPHT	0.1251	0.024	h^{-1}
k15	Partial Vmax for phosphate efflux by PHO1	0.0546	0.025	h^{-1}
k16	km for phosphate efflux by PHO1	0.1174	0.024	μM
β	Flux of miR399 from shoot to root	0.1682	0.02	h^{-1}

** - s.d. is not available for k7 and k8 as they were indirectly calculated from the estimated Y1 and Y2 using the relationship described in the SI.2.

Supplementary Table 4: List of the initial and estimated parameters from each round of model fitting.

Parameters	P1	E1	P2	E2	P3	E3	P4	E4	P5	E5	P6	E6	P7	E7	P1_sd	E1_sd	P2_sd	E2_sd	P3_sd	E3_sd	P4_sd	E4_sd	P5_sd	E5_sd	P6_sd	E6_sd	P7_sd	E7_sd
m1	1	0.87651	0.87651	0.61043	0.61043	0.60586	0.61043	0.4088	0.4088	0.34654	0.34654	0.4017	0.4017	0.42208	1	0.00056	1	0.00355	1	0.02989	1	0.00241	1	0.0147	0.1	0.01573	0.1	0.00359
m2	1	0.14548	0.14548	0.11147	0.11147	0.09069	**0.2**	0.1765	**0.2**	0.2	0.2	0.2	0.2	0.2	1	0.00032	1	0.00023	1	0.00063	1	0.00003	1	0.00105	0.1	0.00104	0.1	0.00099
m3	1	1.4995	1.4995	1.60112	1.60112	3.0102	1.60112	2.2067	2.2067	3.2233	3.2233	3.61274	3.61274	3.5318	1	0.0024	1	0.00718	1	0.00363	1	0.01514	1	0.01542	0.1	0.02669	0.1	0.00515
m4	1	13.19108	13.19108	17.71357	17.71357	11.32366	6.3	6.91307	6.91307	6.70225	6.70225	6.64339	6.64339	6.63974	1	0.00048	1	0.07343	1	0.00067	1	0.00007	1	0.00008	0.1	0.01573	0.1	0.02066
m6	1	0.0243	0.0243	0.00714	0.00714	0.00442	0.00714	0.00635	0.00635	0.0037	0.0037	0.00313	0.00313	0.00279	1	0.00451	1	0.01263	1	0.012	1	0.01548	1	0.01296	0.1	0.00104	0.1	0.00036
m7	1	1.45629	1.45629	1.46229	1.46229	1.49643	1.46229	0.54305	0.54305	2.66623	2.66623	2.41378	2.41378	2.59092	1	0.05171	1	0.06558	1	0.09959	1	0.00163	1	0.01023	0.1	0.02669	0.1	0.01066
m8	1	90.95821	90.95821	105.0393	**1000**	796.9951	**1000**	680.1963	**1000**	191.1368	**1000**	737.5384	**1000**	693.3273	1	0.00046	1	0.00071	1	0.00176	1	0.00055	1	0.00573	0.1	0.00319	0.1	0.00317
k2	1	0.77592	0.77592	1.29827	1.29827	0.30815	1.29827	0.0712	0.0712	0.00383	0.00383	0.00247	0.00247	0.00199	1	0.02569	1	0.01943	1	0.01248	1	0.00163	1	0.00599	0.1	0.04842	0.1	0.01269
k3	1	2.03011	2.03011	2.54835	2.54835	1.91167	2.54835	1.85615	1.85615	1.20187	1.20187	1.10527	1.10527	1.14666	1	0.01397	1	0.02008	1	0.03735	1	0.0018	1	0.01276	0.1	0.00378	0.1	0.00316
k4	1	0.32085	0.32085	0.14541	0.14541	0.09613	0.14541	0.01564	0.01564	0.00871	0.00871	0.00794	0.00794	0.00814	1	0.00245	1	0.00314	1	0.01309	1	0.01728	1	0.0894	0.1	0.019	0.1	0.06508
k5	1	16.94403	16.94403	18.4973	18.4973	20.18231	24	26.11693	26.11693	28.50022	28.50022	28.83186	28.83186	28.88717	1	0.00279	1	0.00122	1	0.00005	1	0.00013	1	0.00043	0.1	0.00026	0.1	0.00055
k7	1	0.000289	0.000289	0.000236	8.99E-06	1.72E-05	9E-07	2.63E-07	2.63E-07	2.79E-07	2.79E-07	2.71E-07	2.71E-07	2.49E-07	1	2.39E-06	1	2.88E-06	1	0.000214	0.001	0.003525	1	0.002774	0.1	3.06E-05	0.1	3.93E-05
k8	1	8.91E-05	8.91E-05	0.0001	3.82E-06	7.36E-06	3.82E-07	1.11E-07	1.11E-07	1.93E-07	1.93E-07	1.92E-07	1.92E-07	1.69E-07	1	4.95E-05	1	3.18E-06	1	1.19E-05	0.001	0.000115	1	9.24E-05	0.1	2.34E-05	0.1	7.55E-05
k9	1	3.72595	3.72595	3.79115	3.79115	3.59343	3.79115	10.36129	10.36129	0.90479	0.90479	0.7801	0.7801	0.76934	1	0.00833	1	0.03601	1	0.25413	1	0.37539	1	0.03612	0.1	0.12679	0.1	0.01784
k10	1	0.26849	0.26849	0.60301	**70**	37.56531	**70**	69.09793	69.09793	38.11847	**70**	74.07617	**70**	64.689	1	0.016	1	0.00764	1	0.00113	1	0.00151	1	0.00105	0.1	0.00278	0.1	0.00735
k11	1	0.65239	0.65239	0.15625	0.15625	0.14065	0.15625	0.12843	0.12843	0.13212	0.13212	0.12018	0.12018	0.12505	1	0.01036	1	0.11416	1	0.06004	1	0.03705	1	0.06518	0.1	0.04051	0.1	0.02384
k15	1	0.05931	0.05931	0.02052	0.02052	0.05858	0.02052	0.06131	0.06131	0.05392	0.05392	0.05744	0.05744	0.05457	1	0.00847	1	0.00932	1	0.09262	1	0.03275	1	0.10101	0.1	0.00195	0.1	0.02492
k16	1	0.5887	0.5887	0.29005	0.29005	0.4711	0.29005	0.17303	0.17303	0.12138	0.12138	0.11616	0.11616	0.11738	1	0.01384	1	0.03604	1	0.14632	1	0.01671	1	0.01725	0.1	0.00728	0.1	0.02365
beta	1	9.25841	9.25841	8.35878	0.1	0.16656	0.16656	0.16786	0.16786	0.16823	0.16823	0.16823	0.16823	0.16823	1	0.00041	1	0.00065	1	0.00537	0.1	0.00156	0.01	0.00041	0.1	0.0126	0.1	0.04975

This table summarises all the used initial values and the corresponding parameter estimates from each round of model fitting. Initial values (P) and parameter estimates (E) for model fitting round (estimation run) 1 to 8. Suffix _sd denotes the corresponding standard deviation for the initial and estimated parameters. Values in bold are manually modified initial value to achieve appropriate fits.

Supplementary Table 5: Parameter estimates and their corresponding standard deviation (s.d.) for each hypothesis model. N/A means not applicable

Parameters	PiOM	PdTA	PdRP	PsMD	PsTR	PsRA	PiOM_sd	PdTA_sd	PdRP_sd	PsMD	PsTR_sd	PsRA_sd
m1	0.422	0.42	0.42684	0.423	0.42624	0.419	0.004	0.00276	0.00027	0.00217	0.00529	0.00244
m2	0.2	0.198	0.19735	0.198	0.19641	0.198	0.001	0.00038	0.00035	0.00157	0.00065	0.000363
m3	3.532	3.54	3.51576	3.53	3.52692	3.58	0.005	0.00107	0.00098	0.000685	0.00192	0.0097
m4	6.639	6.56	6.52457	6.65	6.52561	6.74	0.021	0.00154	0.00057	0.000773	0.00163	0.00116
m6	0.003	0.00278	0.00274	0.0028	0.0028	0.00284	0.0004	0.00042	0.00612	0.00366	0.00089	0.00112
m7	2.6083	2.58	2.59539	2.65	2.59284	2.59	0.0171	0.00162	0.00236	0.00831	0.0031	0.00106
m8	693.33	698	693.41146	693	695.863	695	0.0032	0.000783	0.00226	0.00155	0.00255	0.000693
k2	0.002	0.00201	0.00201	0.002	0.002	0.00202	0.013	0.00193	0.00118	0.002	0.00047	0.00343
k3	1.147	1.16	1.1542	1.14	1.16149	1.15	0.003	0.00274	0.00257	0.00112	0.00183	0.000648
k4	0.008	0.00803	0.00808	0.0081	0.00804	0.00811	0.065	0.00798	0.00571	0.000375	0.00027	0.00171
k5	28.89	29.3	27.44477	29	27.4901	28.9	0.0006	0.000228	0.00059	0.00121	0.0003	0.000835
k9	0.769	0.764	0.75781	0.762	0.75835	0.761	0.017	0.00204	0.00174	0.00417	0.00181	0.00206
k10	64.69	65	64.7655	64.9	64.5804	65.2	0.0074	0.000558	0.00045	0.00133	0.00007	0.00108
k11	0.1251	0.125	0.12371	0.125	0.1265	0.126	0.024	0.00117	0.00289	0.00264	0.00251	0.00253
k15	0.0546	0.0548	0.05552	0.0538	0.05376	0.0549	0.025	0.000137	0.00287	0.00452	0.00274	0.00699
k16	0.1174	0.118	0.11541	0.117	0.11697	0.116	0.024	0.00255	0.00397	0.000964	0.00075	0.0011
beta	0.1682	0.169	0.16651	0.168	0.16638	0.166	0.02	0.000796	0.00659	0.00165	0.00684	0.0119
k6	0.009	0.00252	0.00785	0.0052	0.00711	0.00768		0.603	0.01633	0.61	0.01813	0.59
d4	0.374	7.956372	2.030412235	0.5022	0.8661	0.799307958		2.05	1.07687	0.00232	0.59541	0.00399
d8	N/A	0.0178	0.04163	1.83	1.38067	N/A	N/A	0.0588	0.01062	0.00501	0.00764	N/A
k17	N/A	N/A	2.14	N/A	N/A	N/A	N/A	N/A	0.00454	N/A	N/A	N/A
k18	N/A	N/A	N/A	992.1	544.1	N/A	N/A	N/A	N/A	2.75	0.56	N/A
k19	N/A	N/A	N/A	0.458	1.86	N/A	N/A	N/A	N/A	0.1	0.11	N/A
k20	N/A	N/A	N/A	N/A	N/A	0.233	N/A	N/A	N/A	N/A	N/A	0.0105
m9	N/A	7.12E-07	1.70E-06	0.554	0.72	N/A	N/A	2.30E-07	4.50E-07	2.00E-07	3.06E-07	N/A
Y1	33178.9	10100	9945.07	10000	10071.4	10000	0.04975	0.002	0.0011	0.00347	0.00336	0.0005
Y2	0.4769	0.2	0.145	0.11	0.5533	0.1	0.013	0.00305	0.00685	0.00123	0.00141	0.0018

Supplementary Table 6: Parameter estimates and their corresponding standard deviation (s.d.) for PiOM and PsTR RNA protection (RP) model. N/A means not applicable

parameter	PiOM-RP	PsTR-RP	PiOM-RP_sd	PsTR-RP_sd
m1	0.42467	0.39981	0.03105	0.09896
m2	0.18314	0.18035	0.00153	0.00307
m3	3.41894	3.75762	0.03735	0.02465
m4	7.51522	7.37237	0.00119	0.00269
m6	0.0031	0.00186	0.0403	0.02154
m7	2.48841	2.6484	0.05588	0.01812
m8	11.54216	11.2	0.0049	0.00737
m9	N/A	0.55473	N/A	0.04173
k2	0.00211	0.00203	0.05489	0.13268
k3	1.0059	1.16091	0.01639	0.00984
k4	0.00823	0.00669	0.02189	0.04078
k5	30.91275	30.40525	0.01234	0.00785
k6	0.009	0.00962	N/A	0.01085
k9	0.85494	0.76694	0.03064	0.04294
k10	33.62706	30.0196	0.00438	0.0067
k11	0.12875	0.11749	0.02579	0.06357
k15	0.05582	0.06955	0.02476	0.20403
k16	0.11191	0.11367	0.01394	0.04096
k18	N/A	468.8723	N/A	0.04532
k19	N/A	2.31958	N/A	0.04383
d4	0.375	0.27944	N/A	0.00691
d8	N/A	1.7301	N/A	0.10474
r	2.38675	2.47921	0.00771	0.00449
Y1	1468.106	54.26556	2.82538	6.32246
Y2	0.09089	8.23948	0.04758	0.9399

Supplementary Table 7: Transcription Factor Binding Site prediction for PHO2 upstream region. The upstream 500 bp sequence of OsPHO2 gene (given above) was scan for transcription factor binding site using online tools including PlantPAN (plantpan2.itps.ncku.edu.tw), Jaspar (jaspar.genereg.net) and PROMO (alggen.lsi.upc.es). Top hits with score (>0.8) are presented here.

Tool	Family	Position	Strand	Hit sequence
PlantPAN	bHLH	17	+	accACTTGt
PlantPAN	Homeodomain;TALE	364	+	TGACC
PlantPAN	Myb/SANT	239	+	ctaATATCaa
PlantPAN	Myb/SANT;G2-like	221	+	agAATATtat
PlantPAN	Myb/SANT	239	+	ctaATATCa
PlantPAN	Myb/SANT	292	+	tcATATCta
PlantPAN	Myb/SANT	341	+	cagcATCTTt
PlantPAN	Homeodomain;HD-ZIP	32	+	ctaTTAATga
PlantPAN	MADS box;MIKC;M-type	178	+	tctaaacaaaaggAGAAActc
PlantPAN	MADS box;MIKC;M-type	121	+	ggtctatagaaaaAGAAAaaa
PlantPAN	MADS box;MIKC	181	+	aaaCAAAAggagaaa
PlantPAN	bHLH	425	-	aCAAGTggc
PlantPAN	WRKY	362	-	cTTGACca
PlantPAN	WRKY	361	-	acTTGACcac
PlantPAN	MADF	466	-	gaGTAAAaaa
PlantPAN	WRKY	362	-	cTTGACca
PlantPAN	WRKY	361	-	acTTGACcac
PlantPAN	MADF	466	-	gaGTAAAaaa
PlantPAN	Myb/SANT;G2-like	221	-	agaATATTat
PlantPAN	WRKY	362	-	cTTGACca
PlantPAN	WRKY	360	-	aacTTGACcac
PlantPAN	WRKY	362	-	cTTGACca
PlantPAN	Homeodomain;HD-ZIP	31	-	actATTAAtga
PlantPAN	Homeodomain;HD-ZIP	355	-	ccATTAActt
PlantPAN	WRKY	362	-	cTTGACca
PlantPAN	WRKY	358	-	ttaacTTGACcac
PlantPAN	Myb/SANT	240	-	taATATCaa
PlantPAN	WRKY	363	-	TTGACcactaa
Jaspar	BHLH112	17	+	accacttgt
Jaspar	SPL8	23	+	tgttttgtactattaa
Jaspar	EDT1	32	+	ctattaatga
Jaspar	ATHB-6	106	+	tcaataaat
Jaspar	CDF2	137	+	aaaaaagcca
Jaspar	DOF5.6	137	+	aaaaaagc
Jaspar	DOF5.7	137	+	aaaaaagc
Jaspar	CDF3	138	+	aaaaagcca
Jaspar	Dof2	140	+	aaagcc
Jaspar	Dof3	140	+	aaagcc

Jaspar	MNB1A	140	+	aaagc
Jaspar	PBF	140	+	aaagc
Jaspar	ARR1	291	+	atcatatcta
Jaspar	MYB24	397	+	cagttaggc
Jaspar	MYB46	399	+	gttaggca
Jaspar	MYB59	399	+	gttaggca
Jaspar	ATHB-6	71	-	ttaattata
Jaspar	KAN1	222	-	taatattc
Jaspar	MYB59	236	-	attaggtt
Jaspar	MYB24	237	-	atattaggt
Jaspar	WRKY18	361	-	gtggtcaagt
Jaspar	WRKY8	361	-	tggtcaagt
Jaspar	WRKY2	362	-	tggtcaag
Jaspar	WRKY25	362	-	tggtcaag
Jaspar	WRKY40	362	-	tggtcaag
Jaspar	WRKY63	362	-	tggtcaag
Jaspar	BHLH112	425	-	gccacttgt
Jaspar	NAC043	482	-	aaaacgtaat
PROMO	MYB2	167	+	AACC
PROMO	MYB2	193	+	AACT
PROMO	MYB2	207	+	AACT
PROMO	MYB2	235	+	AACC
PROMO	MYB2	256	+	AACG
PROMO	MYB2	325	+	AACG
PROMO	MYB2	350	+	AGTT
PROMO	MYB2	359	+	AACT
PROMO	MYB2	397	+	AGTT
PROMO	MYB2	411	+	CGTT
PROMO	MYB2	462	+	AACG
PROMO	MYB2	485	+	CGTT

Supplementary Table 8: List of primers used for qRT-PCR	
Primer name	Sequence (5'-3')
PHO2-F	CGAGAATTTTGTCAAGGAGCA
PHO2-R	TCACGAGCATGTCCAACAA
IPS1-F	TTGGCAATTATTCGGTGGAT
IPS1-R	ACCATTTCACCATCCTCTTTATG
Actin-F	CAACACCCCTGCTATGTACG
Actin-R	CCAACACAATACCTGTGGTACG
