# Supplementary material for: Regulatory feedback response mechanisms to phosphate starvation in rice
Source: NPJ Syst Biol Appl. 2018 Jan 8;4:4. doi: 10.1038/s41540-017-0041-0 (PMC5758793; doi:10.1038/s41540-017-0041-0)
Supplement: Supplementary file 3 — Supplementary Figures [file 41540_2017_41_MOESM3_ESM.pdf]

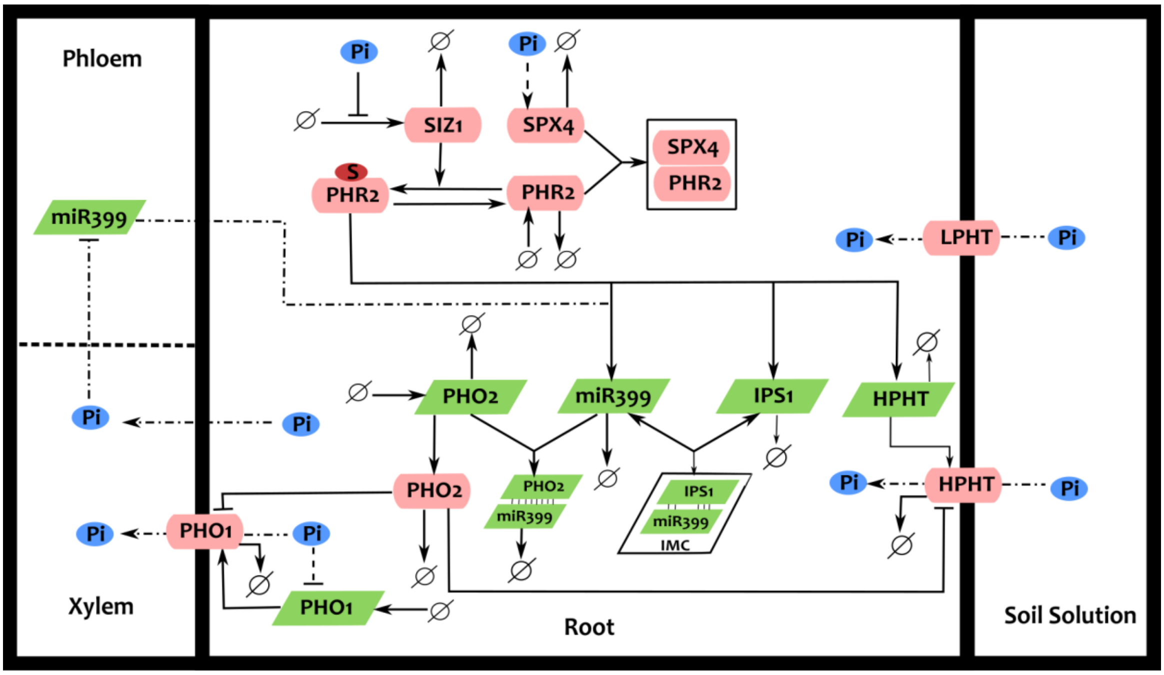

Supplementary Figure 1: Molecular regulation of Pi uptake in plants. Detailed molecular network regulating Pi acquisition. Thick black boxes represent different compartments i.e. soil-solution, cell and vasculature (including phloem and xylem). Protein, ligand, mRNA, complexes and Pi ions are denoted by rectangles (pink), red ellipse, parallelogram (green), squares (transparent) and blue ellipse, respectively. Label acronyms are defined in Table 1. Pointed solid arrows denote direct interactions and fluxes; barred arrows denote inhibition or repression; dashed lines represent indirect interactions. Ø denotes indigenous production and degradation of the molecules.

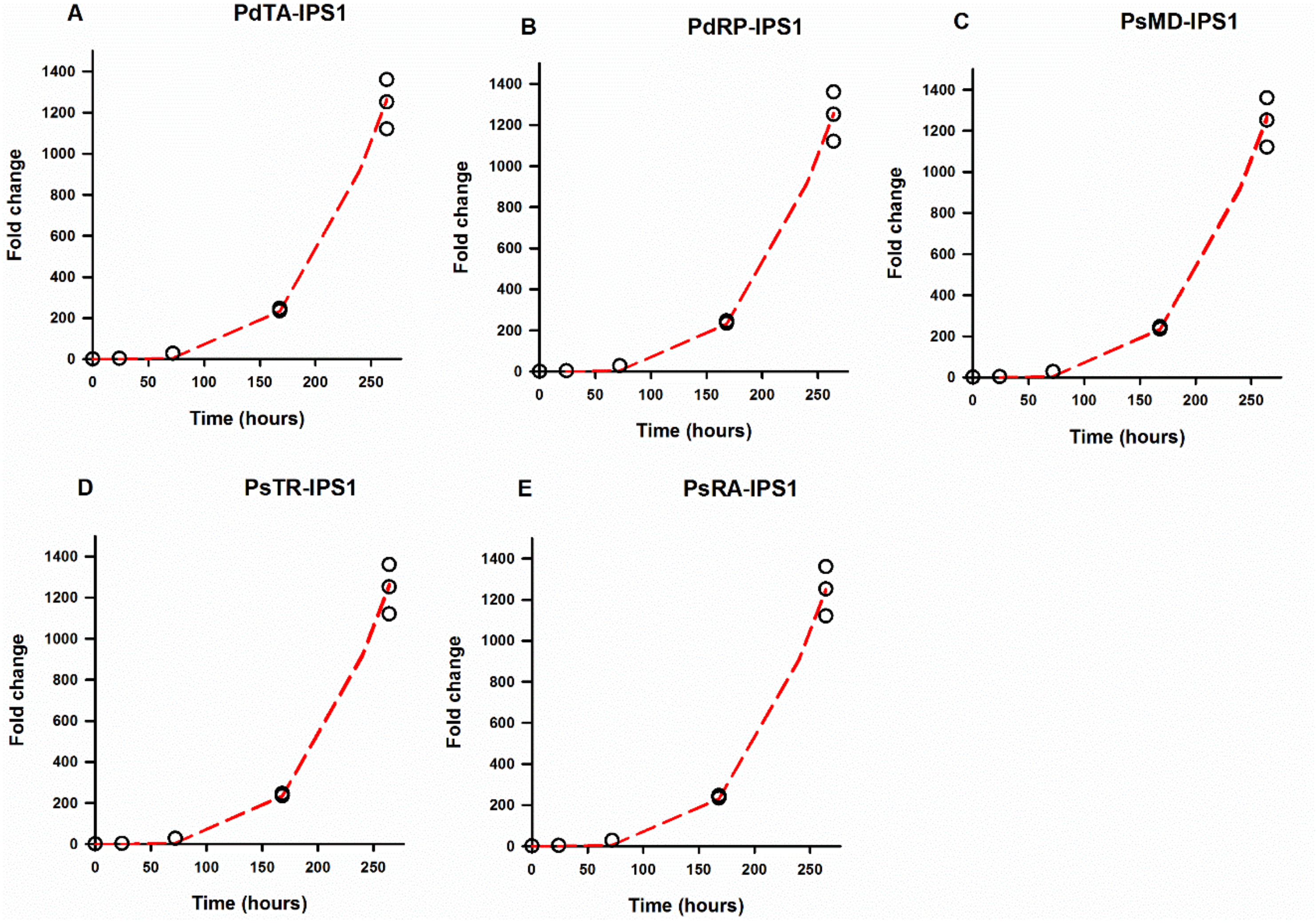

Supplementary Figure 2: All five models show a good fit to IPS1 dataset

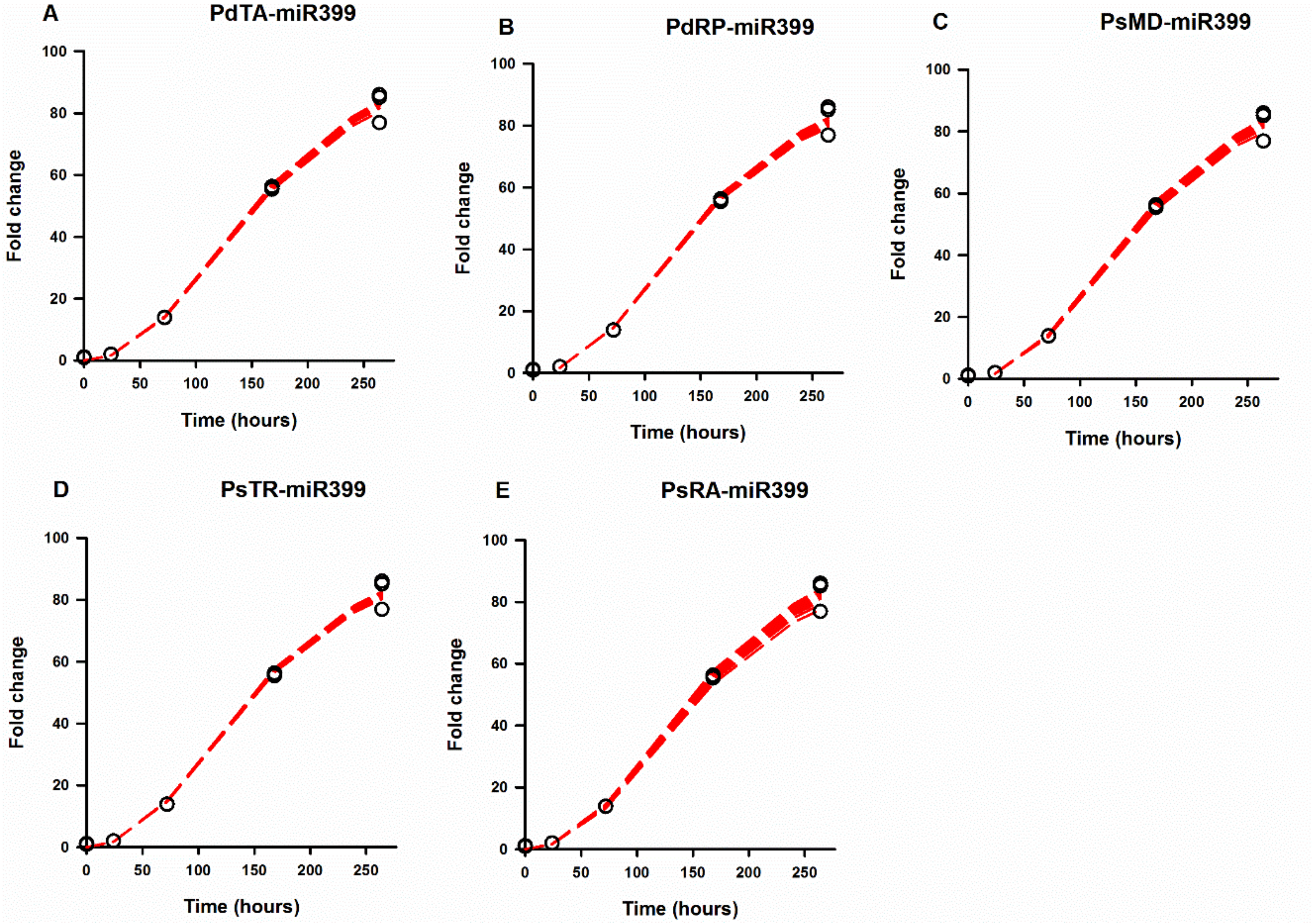

Supplementary Figure 3: All five models show a good fit to miR399 dataset

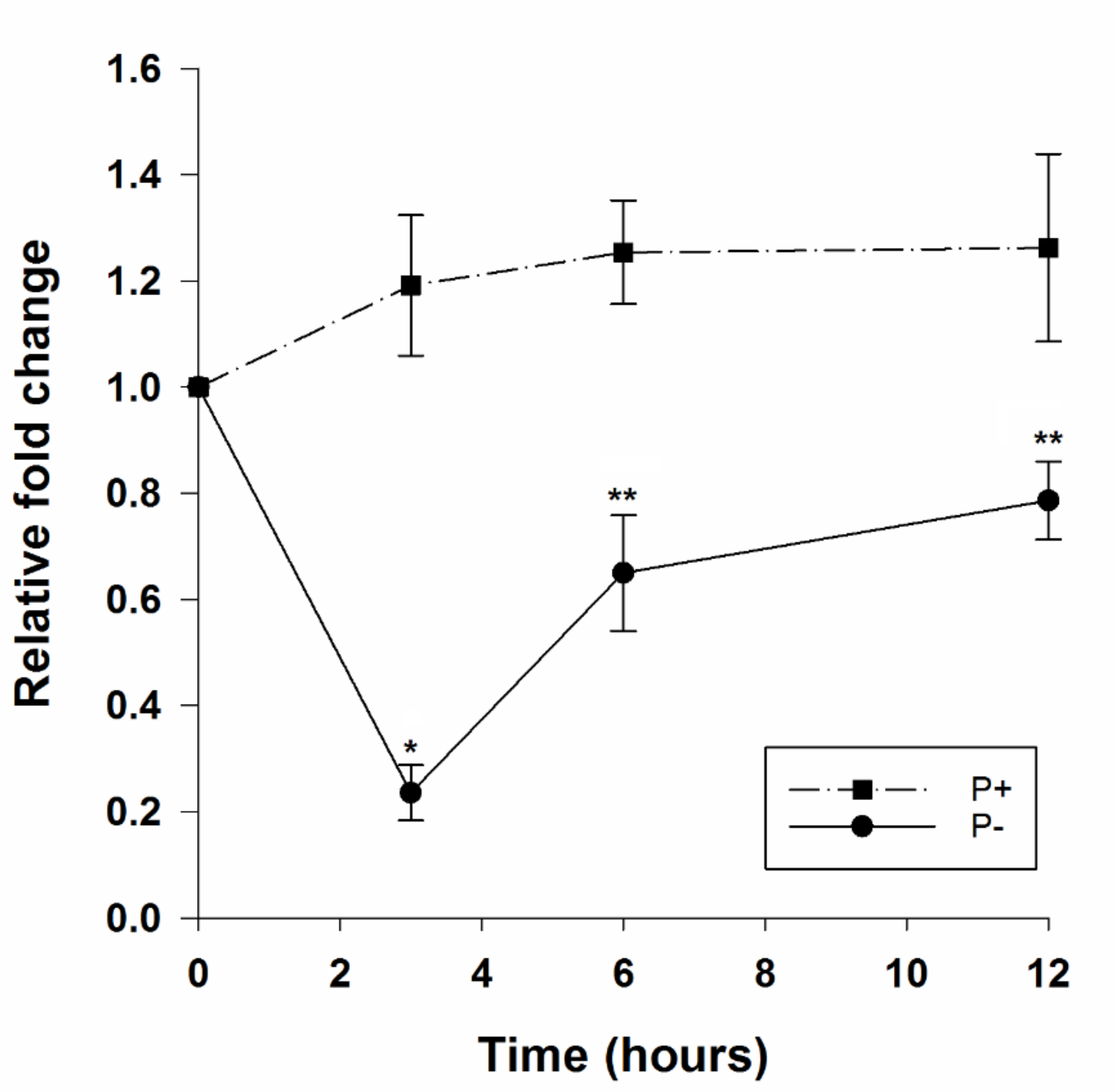

Supplementary Figure 4: Relative fold change in the levels of PHO2 transcript in response to Pi starvation for initial 24 hours. The expression levels are relative to the +P condition at initial time point zero. Errors bars are SE and n = 3. Data significantly different from the corresponding controls are indicated (*P < 0.01; **P < 0.05 Student's t test in Microsoft Excel). Relative expression levels were normalized to that of an internal control, Os-ACTIN.

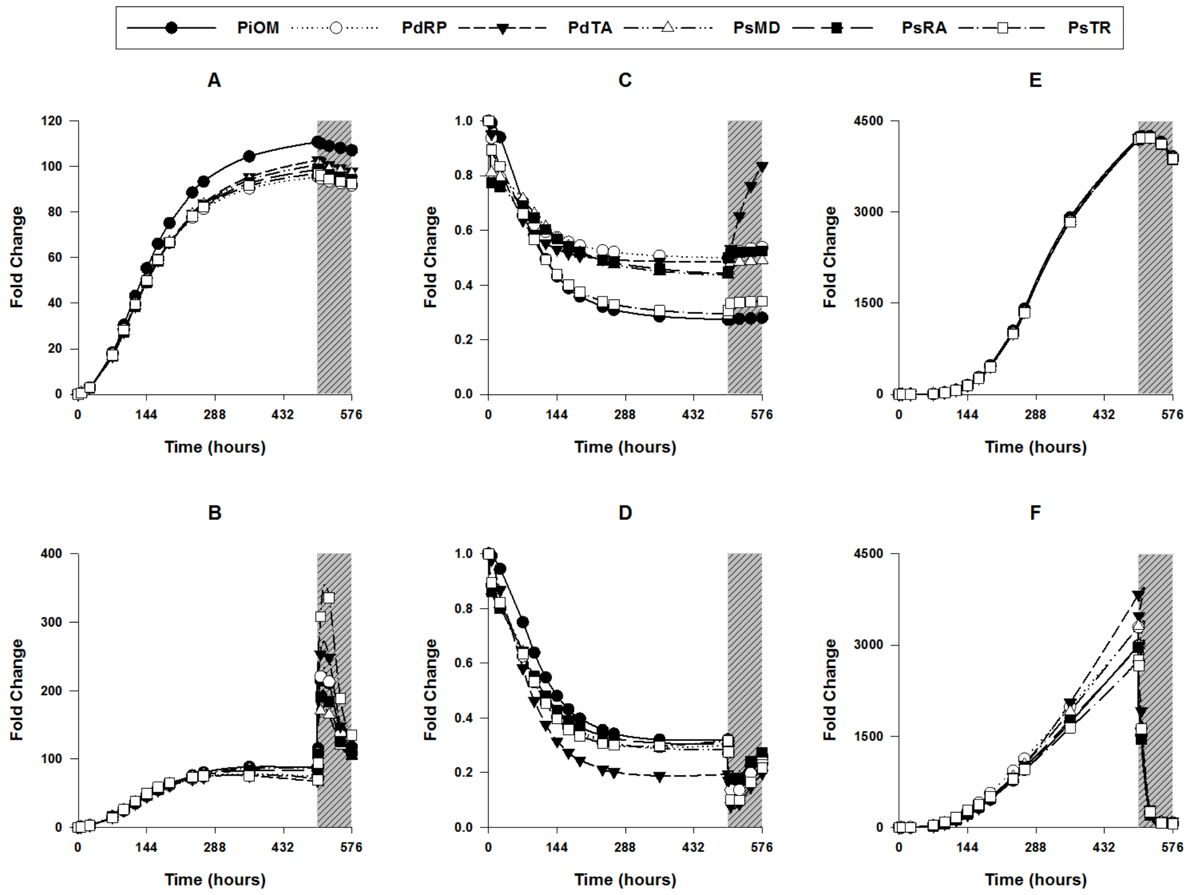

Supplementary Figure 5: Predicted profiles of (A-B) miR399, (C-D) PHO2 and (E-F) IPS1 under Pi-stress and repletion condition from all models (B,D,F) with and (A,C,E) without RNA protection.

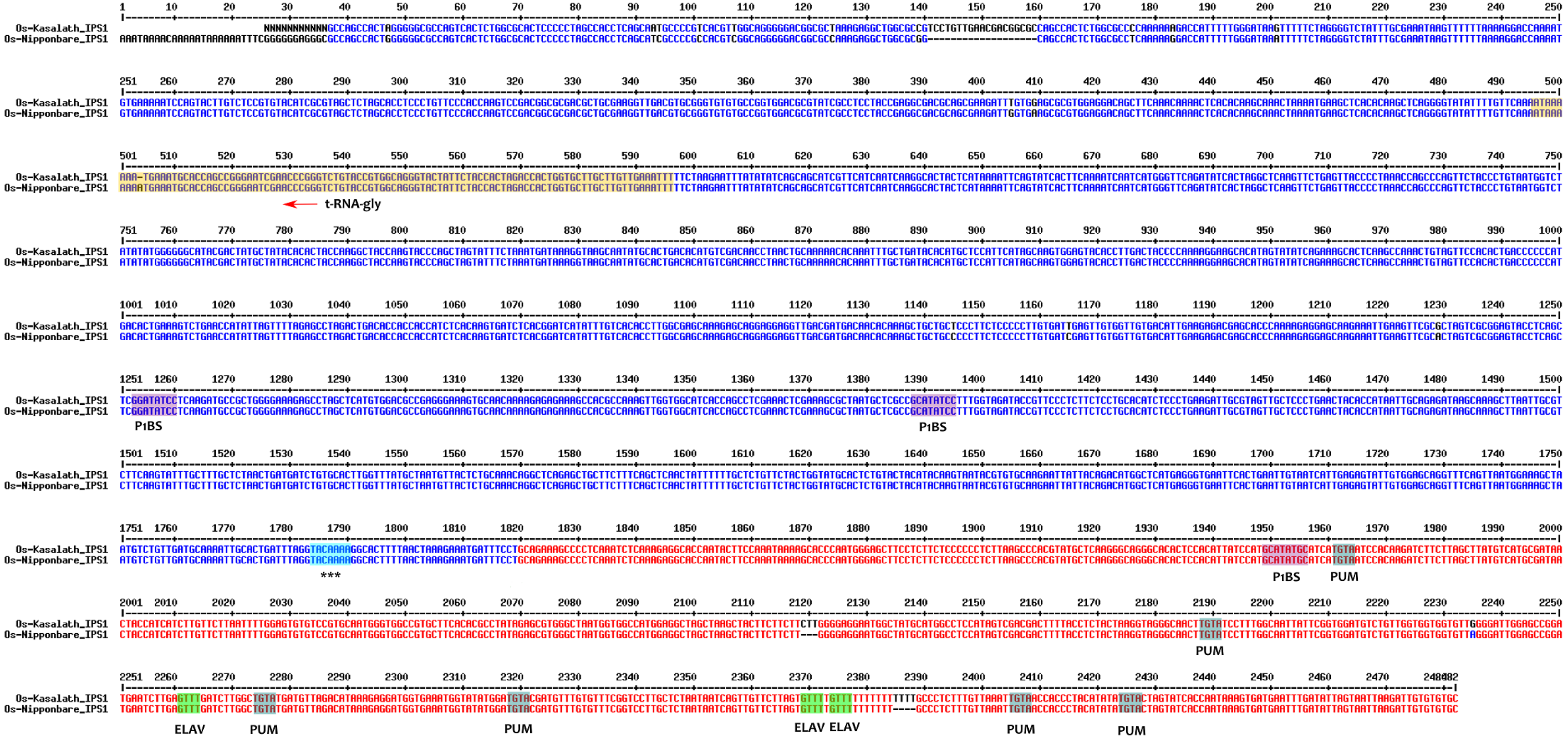

Supplementary Figure 6: Multiple sequence alignment of IPS1 gene and its upstream regulatory region. Red, blue and black text respectively corresponds to IPS1 gene, its upstream regulatory region and insertions in the Kasalath IPS1 sequence. Putative PHR2, Pumilio and ELAV-1 binding sites have been labeled P1BS, PUM and ELAV, and respectively shaded purple, blue-grey and green. The t-RNA-gly sequence and orientation has been labeled and shaded orange. The region shaped in sky blue with stars corresponds to predicted core promoter element.

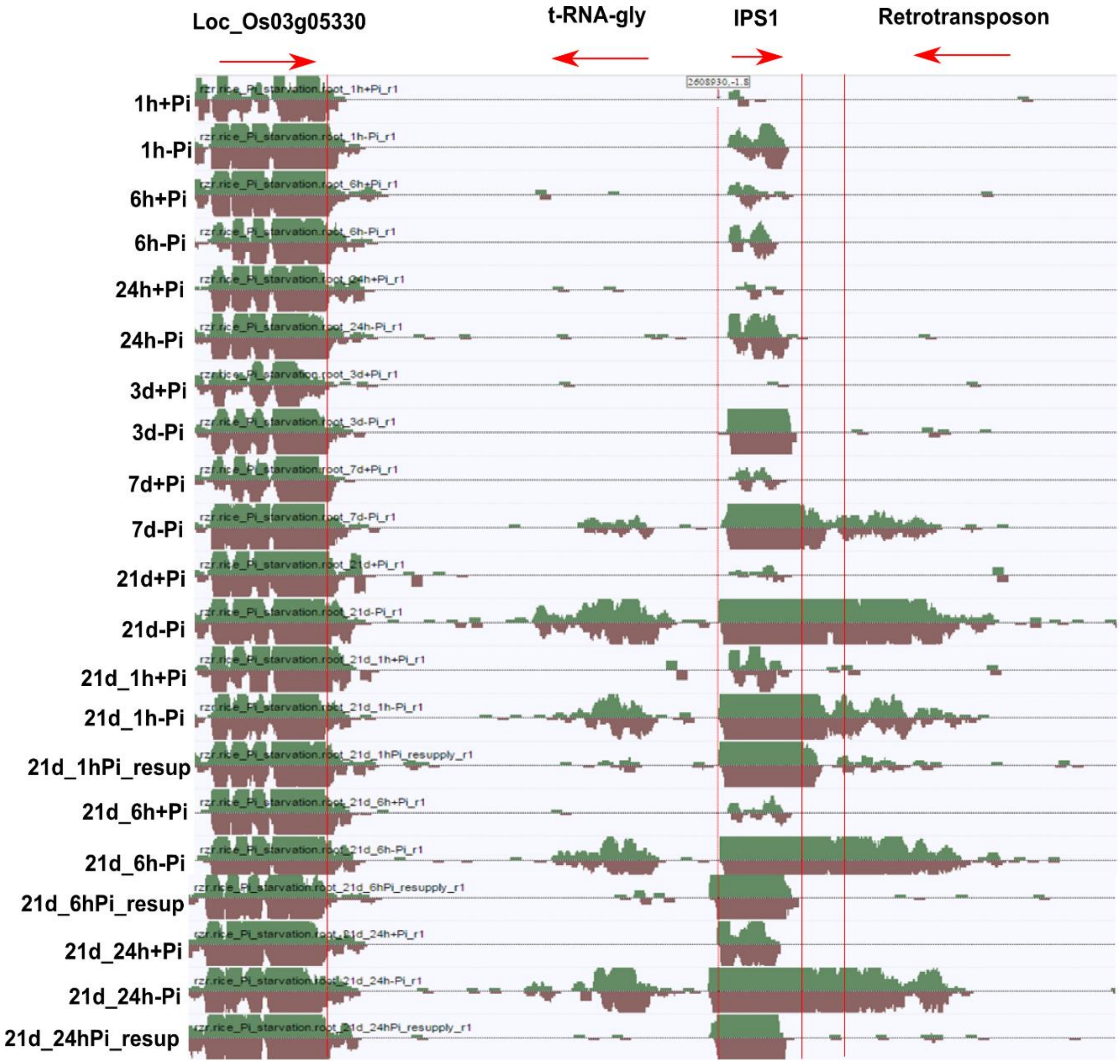

Supplementary Figure 7: Expression regions around the IPS1 locus under normal, deficient and repleted Pi-conditions. A screen capture from the AnnoJ genome browser, representing the expression tracks of IPS1 locus and nearby genes (upstream and downstream) at different time-points under Pi sufficient, deficient and repletion conditions (plantenergy. uwa.edu.au/annoj/Secco_2013.html). The genes corresponding to respective tracks are denote on the top the image along with red arrow depict the direction of transcription.

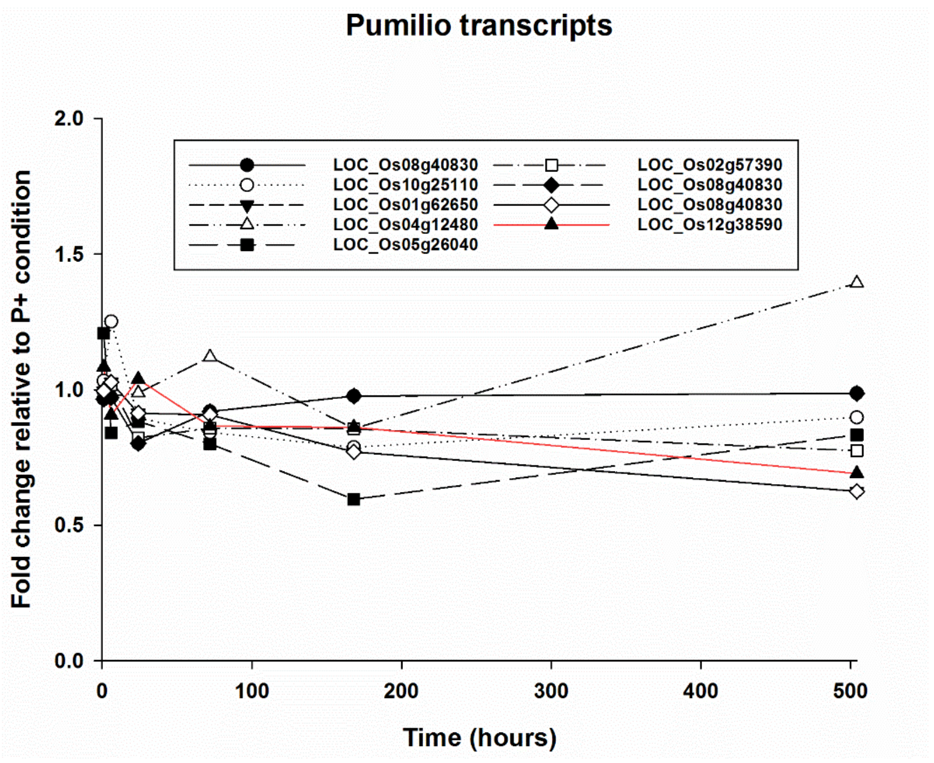

Supplementary Figure 8: mRNASeq expression profiles of different Pumilio genes under Pi-deficient condition

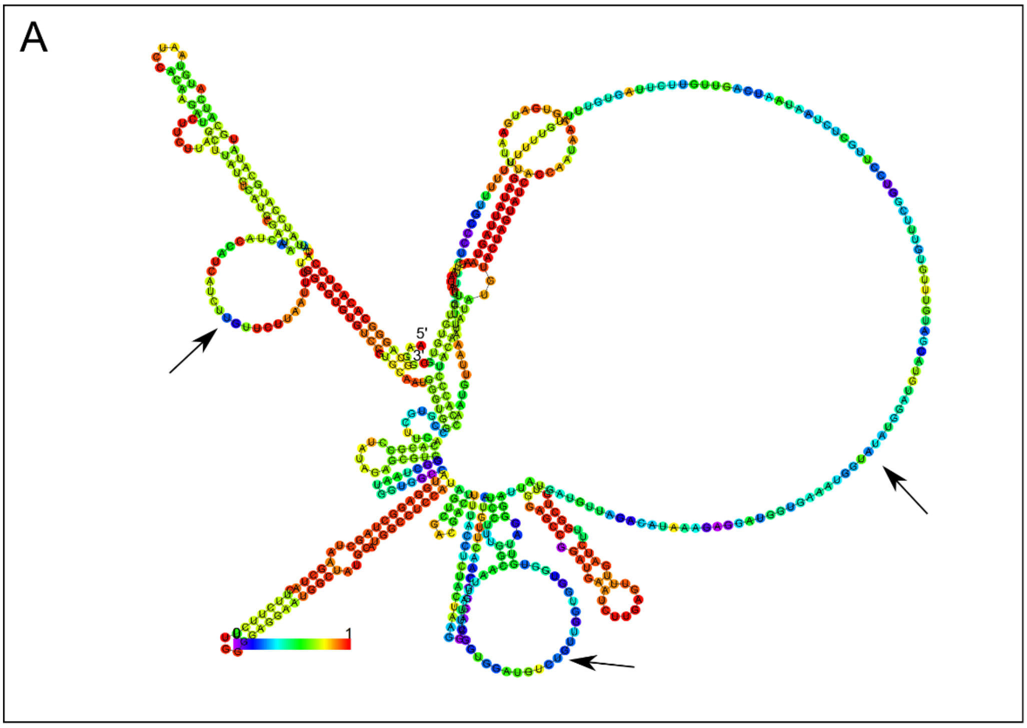

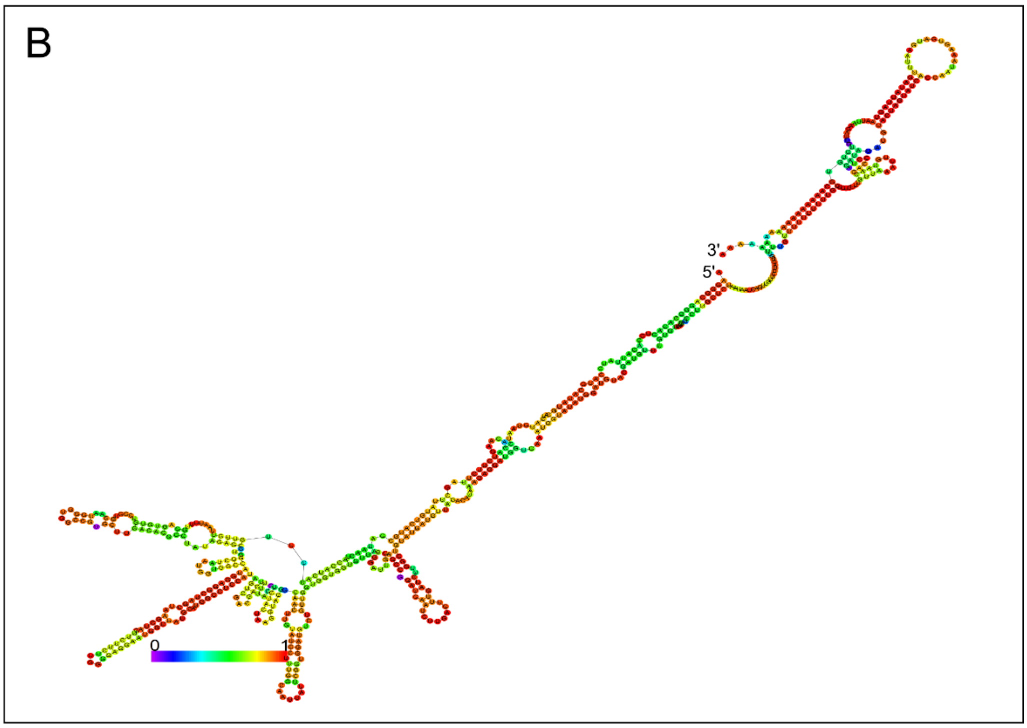

Supplementary Figure 9: Centroid secondary structure prediction of IPS1 mRNA. (A) IPS1 RNA without and (B) with a Poly A tail of 18 bases. The colour scale represents the probability among the ensemble of structures above the minimum free energy structure that the base adopts to form the shown secondary structure. From red to violet denotes most to least probability. Arrows denote RNase sensitive sites.

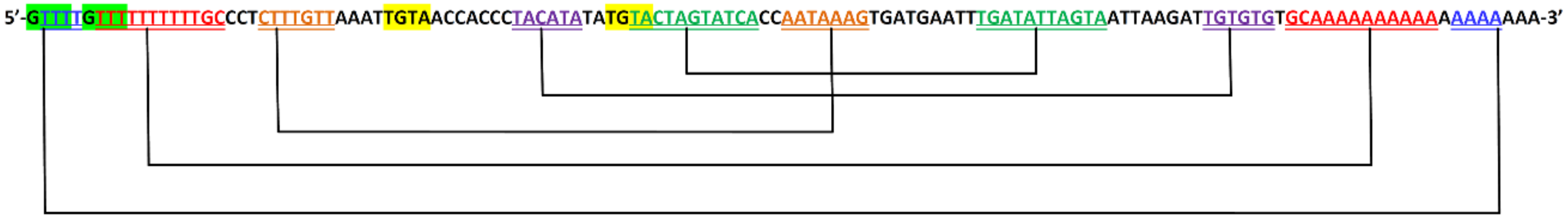

Supplementary Figure 10: Potential pseudoknot near 3’ end of IPS1. The black lines show potential base-pairing structures near 3’ end of IPS1, with the knot shown in purple. The polyA tail could bind to 4 and 12 bases, shown respectively in red and dark blue. The predicted Pumilio and ELAV-1 protein binding sites are highlighted in yellow and green respectively.

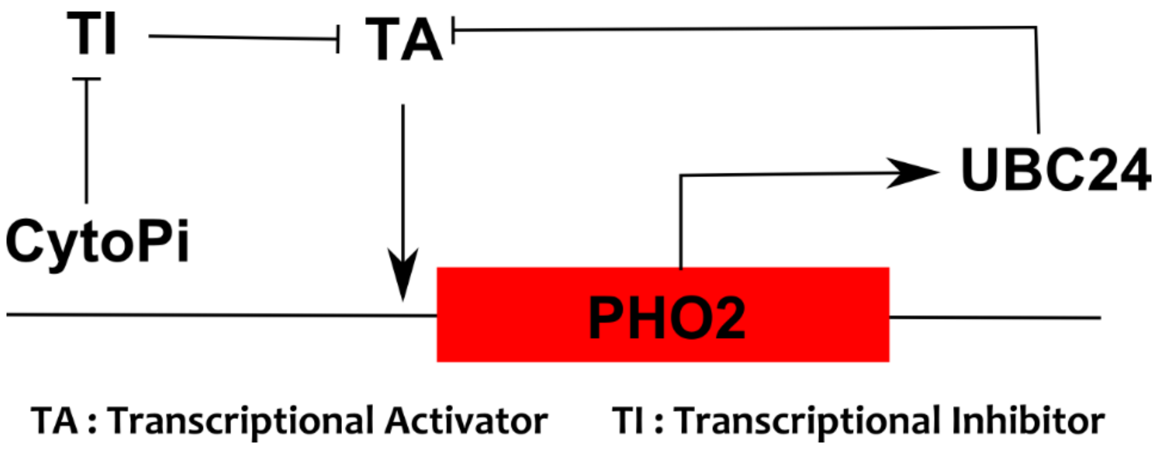

Supplementary Figure 11: Potential model for PHO2 regulation under phosphate deficient condition. PHO2 is expressed courtesy of a Transcriptional Activator (TA), whose degradation it mediates to maintain constant PHO2 levels. However, TA binding efficiency (and hence rate of transcription) is impaired by the binding of a Transcriptional Inhibitor (TI) or Transcriptional repressor (Z) (see Figure 2E), which can only act when Pi is low. Mostly likely TA is a transcription factor that could undergo ubiquitination by PHO2 (UBC24) protein. While the early drop and partial recovery of PHO2 transcript following Pi stress indicate the instantaneous interaction between TA and TI.

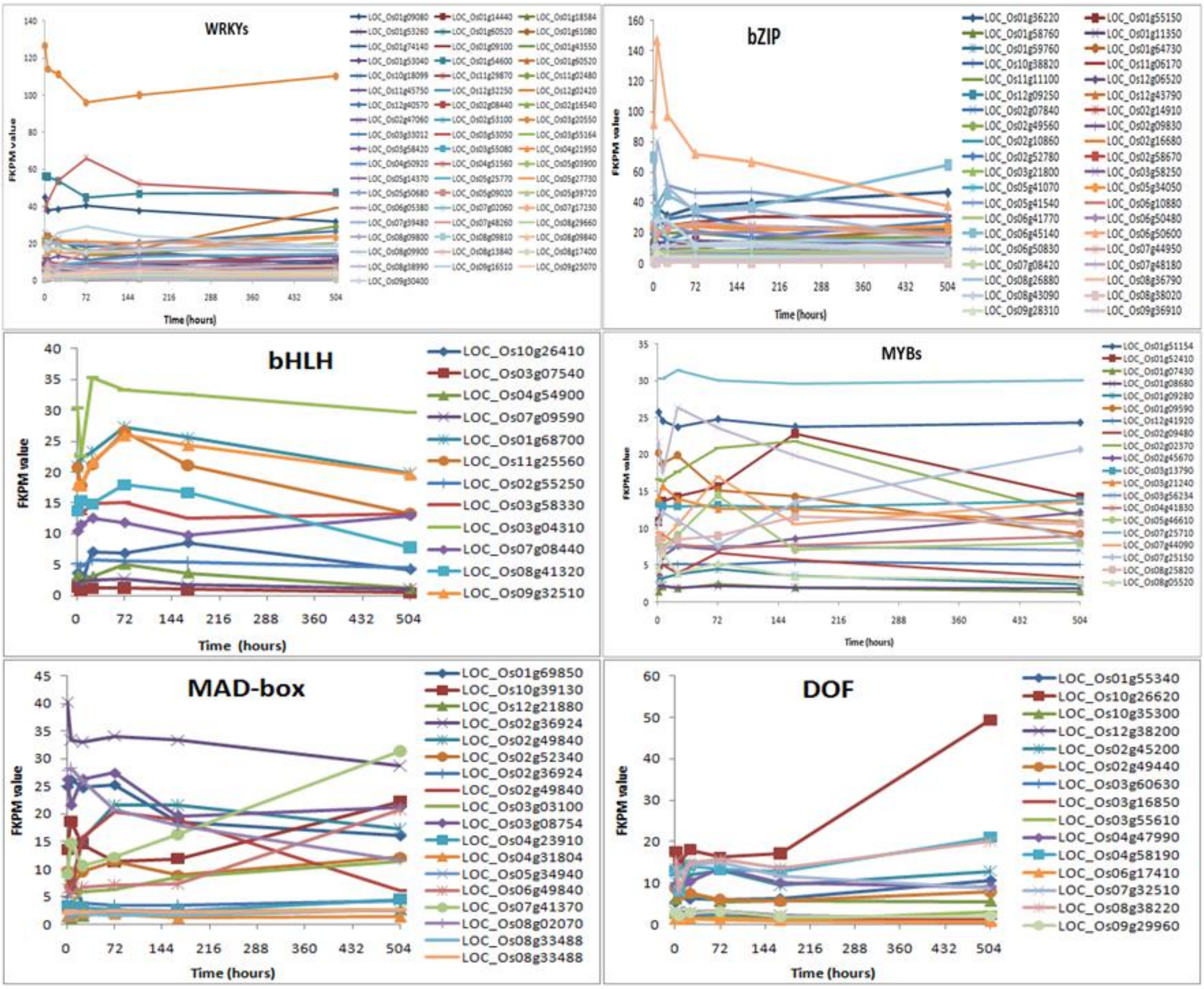

Supplementary Figure 12: mRNAseq expression profile of the members of the transcription factor families under Pi stress. This the transcription factor families are predicted to have binding site in the upstream promoter region of PHO2.

```
>OsIPS1_3'end_69bp_polyA_20bp
cctacatatatgtactagtatcaccaataaagtgatgaatttga
tattagtaattaagattgtgtgtgcaaaaaaaaaaaaaaaaaaaa

ALIGNMENTS
>gnl|SRA|SRR1036340.10531799.1 SRX336041
Length=51

 Score = 75.2 bits (82),  Expect = 7e-13
 Identities = 41/41 (100%), Gaps = 0/41 (0%)
 Strand=Plus/Plus

Query  47  TTAGTAATTAAGATTGTGTGTGCaaaaaaaaaaaaaaaaaa  87
           |||||||||||||||||||||||||||||||||||||||||
Sbjct  1   TTAGTAATTAAGATTGTGTGTGCAAAAAAAAAAAAAAAAAA  41


>gnl|SRA|SRR1036340.19013208.1 SRX336041
Length=51

 Score = 69.8 bits (76),  Expect = 3e-11
 Identities = 38/38 (100%), Gaps = 0/38 (0%)
 Strand=Plus/Plus

Query  52  AATTAAGATTGTGTGTGCaaaaaaaaaaaaaaaaaaaa  89
           ||||||||||||||||||||||||||||||||||||||
Sbjct  1   AATTAAGATTGTGTGTGCAAAAAAAAAAAAAAAAAAAA  38


>gnl|SRA|SRR1036340.13317132.1 SRX336041
Length=51

 Score = 69.8 bits (76),  Expect = 3e-11
 Identities = 38/38 (100%), Gaps = 0/38 (0%)
 Strand=Plus/Plus

Query  45  TATTAGTAATTAAGATTGTGTGTGCaaaaaaaaaaaaa  82
           ||||||||||||||||||||||||||||||||||||||
Sbjct  1   TATTAGTAATTAAGATTGTGTGTGCAAAAAAAAAAAAA  38

>gnl|SRA|SRR1036340.14470642.1 SRX336041
Length=51

 Score = 66.2 bits (72),  Expect = 4e-10
 Identities = 36/36 (100%), Gaps = 0/36 (0%)
 Strand=Plus/Plus

Query  49  AGTAATTAAGATTGTGTGTGCaaaaaaaaaaaaaaa  84
           ||||||||||||||||||||||||||||||||||||
Sbjct  1   AGTAATTAAGATTGTGTGTGCAAAAAAAAAAAAAAA  36


>gnl|SRA|SRR1036340.4553064.1 SRX336041
Length=51

 Score = 64.4 bits (70),  Expect = 1e-09
 Identities = 35/35 (100%), Gaps = 0/35 (0%)
 Strand=Plus/Plus

Query  52  AATTAAGATTGTGTGTGCaaaaaaaaaaaaaaaaa  86
           |||||||||||||||||||||||||||||||||||
Sbjct  1   AATTAAGATTGTGTGTGCAAAAAAAAAAAAAAAAA  35
```

Supplementary Figure 13: BLAST alignment output from querying 69 bp sub-sequence from 3'end of IPS1 concatenated with 20 bp polyA tail against NCBI-Sequence Read Archive (SRX336041). The SRA dataset correspond to the reads from the root starved for 21 days.

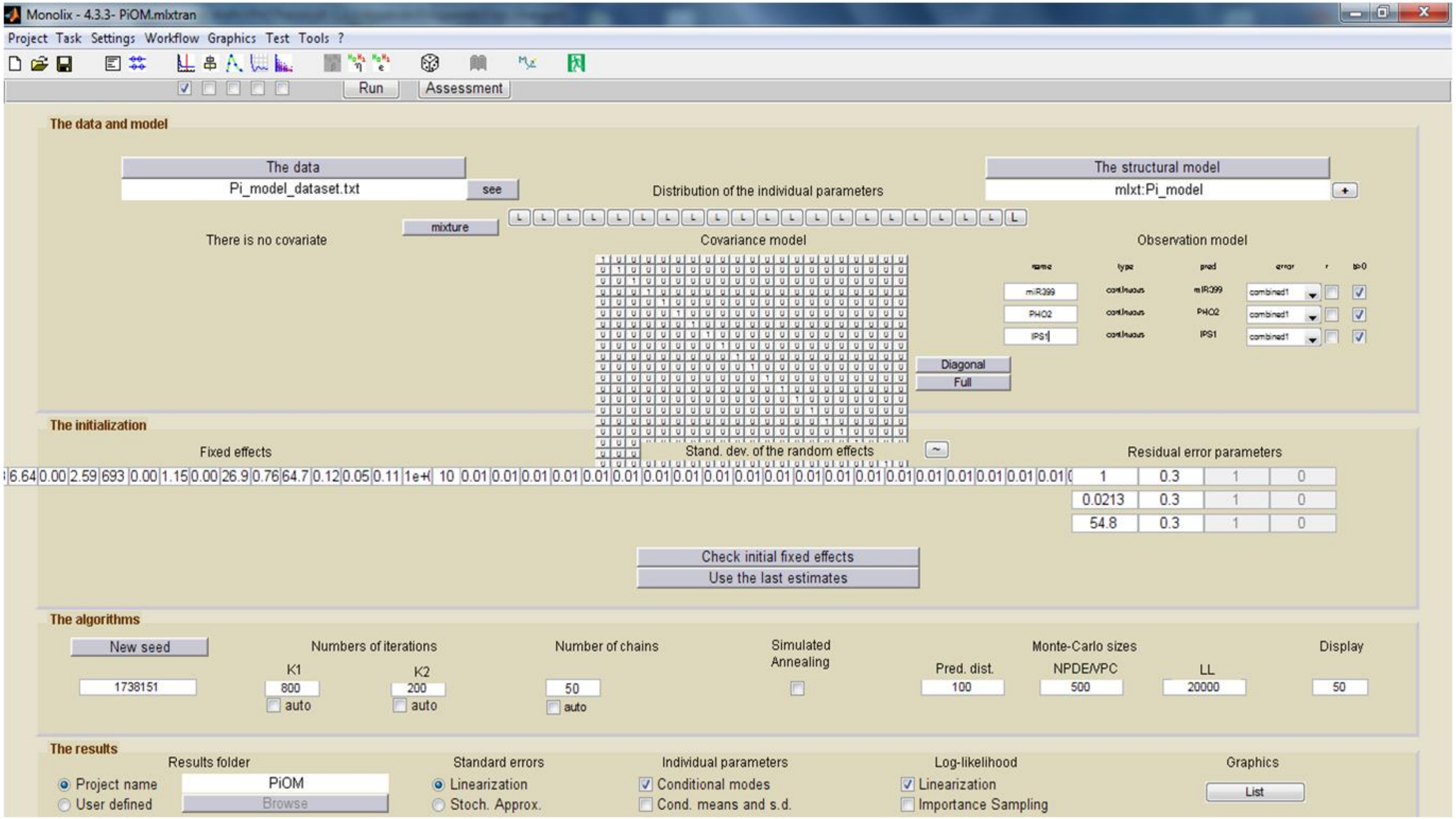

Supplementary Figure 14: Screen capture of MONOLIX platform
